# A FLOE-related protein regulates the two-dimensional to three-dimensional growth transition in the moss *Physcomitrium patens*

**DOI:** 10.1242/dev.204508

**Published:** 2025-08-26

**Authors:** Zoe Weeks, Gargi Chaturvedi, Emily Day, Steven Kelly, Laura A. Moody

**Affiliations:** Department of Biology, University of Oxford, South Parks Road, Oxford OX1 3RB, UK

**Keywords:** 3D growth, Plant development, Apical cell, *Physcomitrium patens*

## Abstract

The colonization of the land by plants coincided with the evolution of three-dimensional (3D) growth: the acquisition of apical cells with the capacity to rotate the plane of cell division. The moss *Physcomitrium patens* has recently been developed as a model system in which to dissect the genetic basis of 3D growth, a unifying feature of all land plants. The cytokinin-unresponsive *nog1-R* mutant incorrectly orients division planes in developing buds and thus fails to make the transition to 3D growth. To reveal the genetic interactors of the *NOG1* gene, which encodes a protein with a C-terminal UBA domain, we performed a screen and identified the *suppressor of nog1a* (*snog1a*) mutant. We have mapped the causative mutation to a gene that encodes a protein related to FLOE2/3 from *Arabidopsis* and demonstrated that the mutant phenotypes observed in both a *nog1* disruptant mutant (*nog1dis*) and *snog1a* can be attributed to changes in cytokinin perception. We present a revised model in which NOG1 operates independently of the APB transcription factors to promote 3D growth initiation.

## Teaser

**Highlighted Article:** The transition to 3D growth is negatively regulated by a FLOE-related protein that alters cytokinin perception and cell division processes in the moss *Physcomitrium patens*.

## INTRODUCTION

In the absence of cell movement, plants rely on cell growth processes, combined with asymmetric and precisely orientated cell divisions, to generate new morphologies and diverse cell types with specialized functions. The main driving force behind cellular diversity is the formation of apical cells that can divide to self-renew and generate new cell types, and it is the geometry of an apical cell and the way it divides that can greatly influence the pattern of growth and development that follows. Thus, diversification of plant form can largely be attributed to altered division processes in apical cells, which occurred before the transition from water to land ∼470 million years ago (Kenrick and Crane, 1997).

Water-dwelling charophycean green algae can develop apical cells, but these only have the capacity for either one-dimensional (1D) growth (one cutting face) or two-dimensional (2D) growth (two cutting faces). As a result, the morphologies represented within the multicellular charophytes are typically filamentous (e.g. *Chara braunii*) or disc-like (e.g. *Coleochaete orbicularis*) (Kenrick and Crane, 1997; Wickett et al., 2014; Delwiche and Cooper, 2015; Harrison, 2017). Three-dimensional (3D) growth, the development of apical cells with three or more cutting faces, is a unifying feature of all land plants. It was likely the emergence of 3D growth processes, along with the development of a multicellular sporophyte and the acquisition of vegetative desiccation tolerance, that enabled successful terrestrialization (Delwiche and Cooper, 2015; Harrison, 2017; Moody, 2020).

The gametophyte of the moss *Physcomitrium patens* is well suited to studies of 3D growth. This is because an extensive 2D filamentous growth phase precedes the transition to 3D growth, and thus the 2D-to-3D growth transition can be studied without causing lethality, as the 2D growth phase can be vegetatively propagated in perpetuity (Moody et al., 2018a,b, 2021). The gametophyte phase of the *P. patens* life cycle begins with the germination of a haploid spore, which gives rise to a 2D branching filamentous network known as the protonema, which extends by tip growth. The protonema consists of two cell types; first to emerge are the chloroplast-dense chloronemal cells, and subsequently caulonemal cells that form because of an auxin-mediated reprogramming of a chloronemal apical cell into a caulonemal apical cell (Jang and Dolan, 2011; Jaeger and Moody, 2021). Lateral protrusions derived from caulonemal subapical cells give rise to side branch initials, most of which go on to form secondary protonema but some acquire 3D fate (gametophore initials) and give rise to gametophores (∼5%) (Aoyama et al., 2012). The fate of a side branch initial is likely determined by highly localized cues within the parental cell before side branch emergence, as a single caulonemal subapical cell can simultaneously divide to give rise to both a filament and a gametophore (Harrison et al., 2009). It has been shown that persistent expression of a group of AP2-type transcription factors (APB1-4) is required to commit a side branch to 3D cell fate. Loss of APB1-4 expression corresponds to the maintenance of 2D cell fate, and consequently, mutants lacking all four genes fail to make the transition from 2D to 3D growth (Aoyama et al., 2012).

A filament initial is readily distinguishable from a gametophore initial: filament initials expand by tip growth and divide parallel to the cell from which they are derived, and gametophore initials swell diffusely and divide in a characteristically oblique manner during the specification of 3D growth, at the first, second and third divisions of the developing gametophore. Successive rotating divisions then lead to the establishment of a tetrahedral apical cell at the apex of the shoot. The apical cell continuously self-renews and divides only from the three downward faces to form phyllid initials that go on to form the leaf-like phyllids that are organized around the central axes of developing gametophores in a spiral phyllotaxy (Harrison et al., 2009). Mature gametophores bear both archegonia and antheridia, which house eggs and sperm, respectively. Following fertilization, a mitotic programme initiates, leading to the formation of a multicellular diploid sporophyte, which undergoes meiosis to produce haploid spores to restart the life cycle (Cove and Knight, 1993).

It has long been known that cytokinin induces the formation of gametophore initials but is insufficient to maintain 3D growth, as treatment with high levels of cytokinin produces buds that develop into callus-like tissue, rather than structurally organized gametophores (Brandes and Kende, 1968; Ashton et al., 1979). In a variety of developmental contexts, auxin has been consistently implicated as the signal required to establish asymmetry in plants (Petricka et al., 2009; Shao and Dong, 2016). Perhaps unsurprisingly, the formation of gametophore initial cells and the specification and maintenance of 3D growth also relies upon auxin signalling, although the application of high levels of auxin can antagonize cytokinin and reverse the effects of cytokinin treatment (Brandes and Kende, 1968). Nevertheless, auxin levels are notably high during the specification of 3D growth but diminish once a tetrahedral apical cell has been established (Thelander et al., 2019). It is likely that auxin acts to promote cell differentiation and that cytokinin acts to continually maintain apical cell proliferation (Hata et al., 2024, 2025). Thus, it appears that a highly regulated balancing act between auxin and cytokinin is required for both the specification and maintenance of 3D growth.

In recent years, functional studies have demonstrated that the transition to 3D growth is complex and regulated at many levels; both epigenetically (Mosquna et al., 2009; Okano et al., 2009; Raquid et al., 2023) and transcriptionally (Aoyama et al., 2012), and an ever-expanding number of studies have begun to connect complex cell signalling pathways to post-translational regulation (Girod et al., 1999; Perroud et al., 2014, 2020; Demko et al., 2014; Johansen et al., 2016; Hoernstein et al., 2016; Schuessele et al., 2016; Whitewoods et al., 2018; Moody et al., 2018a, 2021; Cammarata et al., 2022).

Using a forward genetics approach, we previously demonstrated that the *NO GAMETOPHORES 1* (*NOG1*) gene is essential for the transition to 3D growth in *P. patens* (Moody et al., 2018a). Notably, the *NOG1* gene encodes a protein with a C-terminal ubiquitin-associated (UBA) domain. UBA domains are often involved in protein degradation processes (Hofmann and Bucher, 1996; Su and Lau, 2009; Moody et al., 2018a). Mutants lacking a functional copy of *NOG1* (the ‘*no gametophores 1 – Reference*’ mutant; *nog1-R*) produce significantly fewer gametophore initials than wild type, even in the presence of cytokinin. Those gametophore initial cells that do form cannot correctly orient the characteristically oblique plane of the first cell division. Cell division planes are then misplaced thereafter, leading to the formation of defective gametophores that undergo very early developmental arrest (Moody et al., 2018a). Since the APB genes are downregulated in the *nog1-R* mutant, we previously proposed that NOG1 may positively regulate 3D growth by degrading a repressor of APB transcriptional activation, although the identity of the target(s) remains unknown (Aoyama et al., 2012; Moody et al., 2018a). To build on our understanding of the role played by *NOG1* in both the initiation and specification of 3D growth, we generated a *nog1* disruption mutant (*nog1dis*) and then performed a suppressor screen to identify mutations that alleviated the *nog1dis* mutant phenotype (i.e. reversion to 3D growth). Here, we describe the screen, along with the detailed characterization of the *suppressor of nog1a* (*snog1a*) mutant and the identification of the causative mutation within a gene encoding a FLOE-related protein.

## RESULTS

### Disruption of the *NOG1* locus recapitulates the *nog1-R* mutant phenotype

Previously, we performed a UV-mediated forward genetic screen that led to the identification of the *NOG1* gene (Moody et al., 2018a). To explore the *NOG1* genetic interaction network underpinning the 2D to 3D growth transition, we designed a suppressor screen to identify mutations that alleviated the 3D-defective phenotype caused by loss of *NOG1* function (i.e. the reacquisition of 3D growth). A preliminary suppressor screen of ∼3000 UV-mutagenized lines of the original *nog1-R* mutant, generated in our original forward genetic screen, identified eight mutants that exhibited a complete reversion to 3D growth. In each of these mutants, the phenotype was caused by correction of the previously described mutation: a T>C transition that converted the premature termination codon back to an arginine residue. We therefore decided to disrupt the *NOG1* locus in such a way that would prevent repair simply through the introduction of UV-induced single-nucleotide polymorphisms (SNPs). To that end, we set out to generate a *nog1* knockout mutant in which the entire coding sequence of *NOG1* had been replaced with a hygromycin resistance cassette. Several attempts were made to obtain a line in which the entire *NOG1* sequence had been removed (Fig. S1A). However, as was the case in previous attempts (Moody et al., 2018a), we could achieve disruption of the *NOG1* locus but unfortunately not a full deletion. Nevertheless, a *nog1* null mutant was generated in which expression of the gene was abolished (Fig. S1B). Consistent with the original *nog1-R* mutant, *nog1dis* completely and consistently failed to make the transition to 3D growth (Fig. S1C). Thus, disruption of the *NOG1* locus recapitulated the original *nog1-R* mutant phenotype and generated a suitable line for mutagenesis.

### The *snog1a* mutant can specify 3D growth and responds to cytokinin

**Fig. 1.**
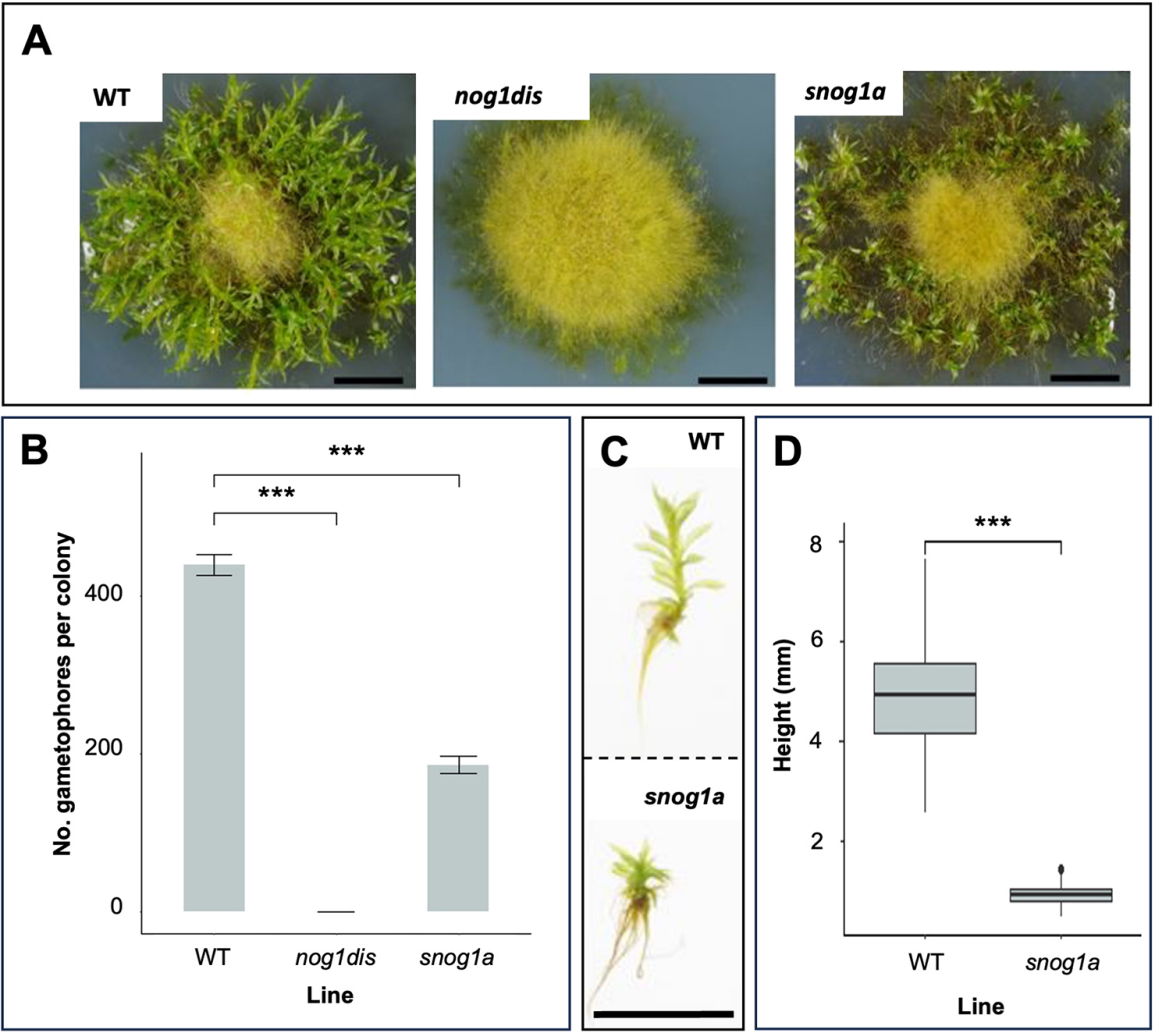
**The *snog1a* mutant exhibits a partial restoration of 3D growth.** (A) Representative images of 6-week-old Villersexel wild-type (WT), *nog1dis* and *snog1a* plants showing the presence (WT and *snog1a*) and absence (*nog1dis*) of gametophores. (B) Mean number of gametophores per culture (*n*=5) ±s.e.m. (two-tailed unpaired Student's *t*-test; ***P<0.05). (C) Representative images of a gametophore from wild type (top) and stunted gametophore from the *snog1a* mutant (bottom). (D) Mean height of gametophores from wild type (*n*=100) and *snog1a* (*n*=80) ±s.e.m. (two-tailed unpaired Student's *t*-test; ***P<0.001). Box plot shows mean values (middle values) and first to third interquartile ranges (boxes); whiskers indicate 1.5x the interquartile ranges; dots indicate outliers. Scale bars: 1 cm (A and C).

A suppressor screen of 2864 UV-mutagenized lines of the *nog1dis* mutant yielded two *suppressor of nog1* (*snog1*) mutants that exhibited a restoration of 3D growth. In one of these mutants, *snog1a*, the formation of gametophores was partially restored to ∼45% of the frequency of wild type in standard growth conditions (Fig. 1A,B), although these were somewhat stunted and emerged later than those formed in the wild type (Fig. 1C,D).

**Fig. 2.**
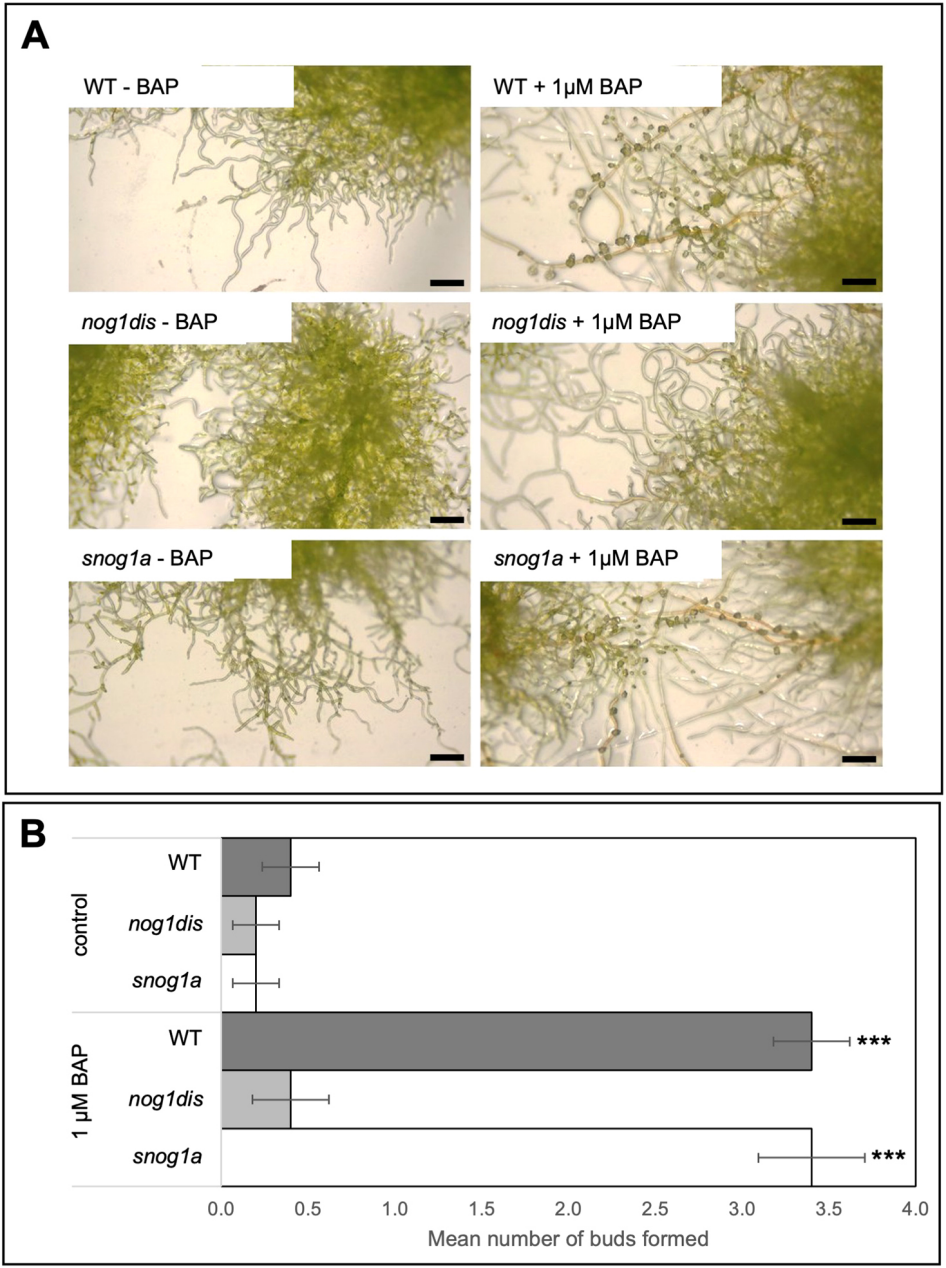
**The *snog1a* mutant is cytokinin responsive.** (A) Representative images of wild-type (WT), *nog1dis* and *snog1a* plants cultured in the presence or absence of the cytokinin analogue 6-benzylaminopurine (BAP). (B) Mean number of buds formed per 15 caulonemal cells in wild type, *nog1dis* and *snog1a* in the presence or absence of BAP (*n*=10) ±s.e.m. (two-tailed unpaired Student's *t*-test; ***P<0.001). Scale bars: 0.5 mm.

In contrast to the *nog1dis* mutant, in which responses to cytokinin were impaired in a similar manner to that of the *nog1-R* mutant, buds were induced by cytokinin treatment in the *snog1a* mutant similarly to wild type (Fig. 2). Thus, the mutant phenotypes observed in both the *nog1dis* and *snog1a* mutants could be attributed to changes in cytokinin perception.

**Fig. 3.**
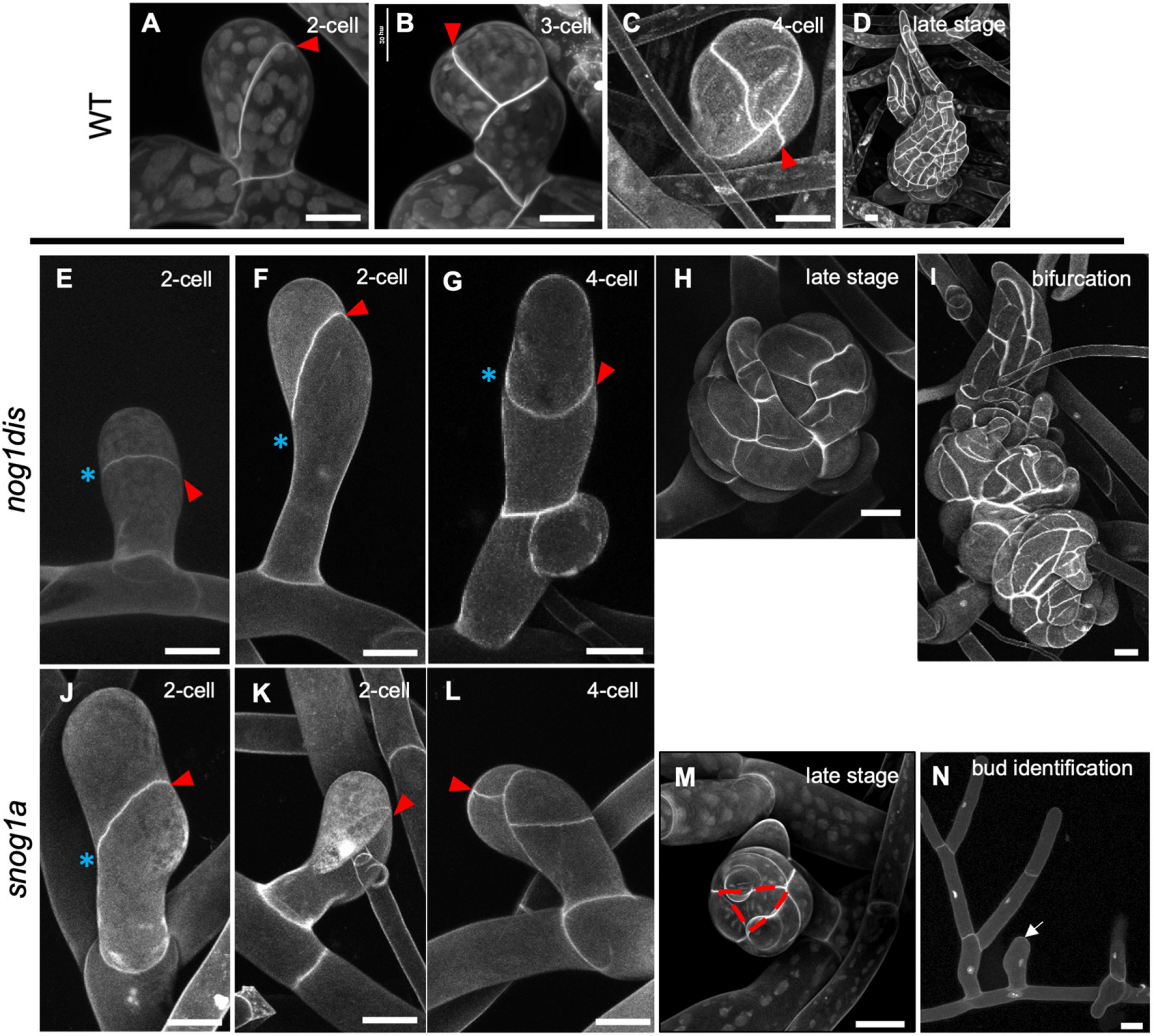
**The *snog1a* mutant can establish and maintain a tetrahedral apical cell.** (A-N) Propidium-Iodide-stained buds of wild type at the two-cell (A), three-cell (B), four-cell (C) and late stage (D); the *nog1dis* mutant at the two-cell (E,F), four-cell (G), late stage (H) and during bifurcation (I); and the *snog1a* mutant at the two-cell (J,K), four-cell (L) and late stage (M). (N) Detection of gametophore initial cells near side branches with filament fate that fail to divide (denoted by white arrow). Red arrows denote the most recent division in each developing bud, and blue asterisks highlight misoriented division planes in *nog1dis* or *snog1a* mutants. Red hashed line denotes the conspicuous tetrahedral-shaped gametophore apical cell formed in *snog1a* in M. Scale bars: 20 µm.

To determine whether the cell division orientation defects had been repaired in the *snog1a* mutant, we obtained *z*-stack projections of developing buds stained with Propidium Iodide. In wild-type *P. patens*, the first division of the gametophore initial cell was invariably oblique and yielded an apical and a basal cell (Fig. 3A). Two additional oblique divisions of the apical and basal cells then occurred rather synchronously. Although the angle of the division plane was consistent in each case, the order in which the cells divided was inconsistent; we generally observed that the apical cell divided before the basal cell as often as the basal cell divided before the apical cell (Fig. 3B,C). Successive rotating divisions specified a gametophore apical cell with a characteristic tetrahedral shape, which self-renewed and divided to give rise to the phyllids, which wrap around the central axis of the gametophore in a spiral phyllotaxy (Fig. 3D). In the *nog1dis* mutant, similar to the previously described *nog1-R* mutant, significantly fewer gametophore initial cells formed, and the first division plane of the gametophore initial cell was not characteristically oblique. In most cases, the initial division pattern followed that of a filament initial cell, in which the division plane was positioned roughly parallel to the parental cell from which it was derived (Fig. 3E). Cell plates were then positioned randomly during subsequent divisions, which prevented the specification and maintenance of a tetrahedral apical cell. In some cases, the gametophore initial cells swelled and elongated excessively, but then divided in a somewhat oblique manner at the first and second divisions (Fig. 3F). However, cell plates were invariably misplaced from the onset of the third division (Fig. 3G). Most developing gametophores arrested early in development, but occasionally callus-like buds appeared because of uncontrolled proliferation (Fig. 3H). Moreover, bifurcation events were occasionally observed, which were a result of confused cell fate acquisition, and often associated with supernumerary apical cell formation (Fig. 3I). However, none of these apical cells was successfully maintained long enough to produce a gametophore. In the *snog1a* mutant, the formation of gametophore initial cells was partially restored, and these largely followed a wild-type pattern of development, with some exceptions. In some cases, we observed that the angle of the first division plane had been corrected to some extent, but not fully. In these examples, it was likely that a mature gametophore would fail to form, given the close resemblance to the *nog1dis* mutant phenotype (Fig. 3J). However, in most cases, the first division plane was characteristically oblique and thus the orientation of the division plane had been fully corrected (Fig. 3K). This was followed by two further correctly oriented oblique divisions (Fig. 3L), which formed the prerequisites for the specification of a conspicuous tetrahedral apical cell at the apex of developing gametophores (Fig. 3M). Thus, restoration of gametophore initial cell formation in the *snog1a* mutant was accompanied by the reversion of the cell division orientation defects observed in the *nog1dis* mutant.

Notably, the phenotype observed in the *snog1a* mutant only constituted a partial restoration of the *nog1dis* phenotype. This could be because the fate of side branch initials formed in the *snog1a* mutant is not fully committed – i.e. it was not unusual to observe bulbous gametophore initial cells that had failed to divide. The proximity of these cells to those side branch initials that had already committed to filament fate served as a reliable marker of cell identity and thus supports this explanation (Fig. 3N). Nevertheless, a tetrahedral apical cell could be established and maintained in the *snog1a* mutant, and the gametophores formed produced viable gametangia.

### The causative mutation of *snog1a* resides in a gene that encodes a FLOE-related protein

**Fig. 4.**
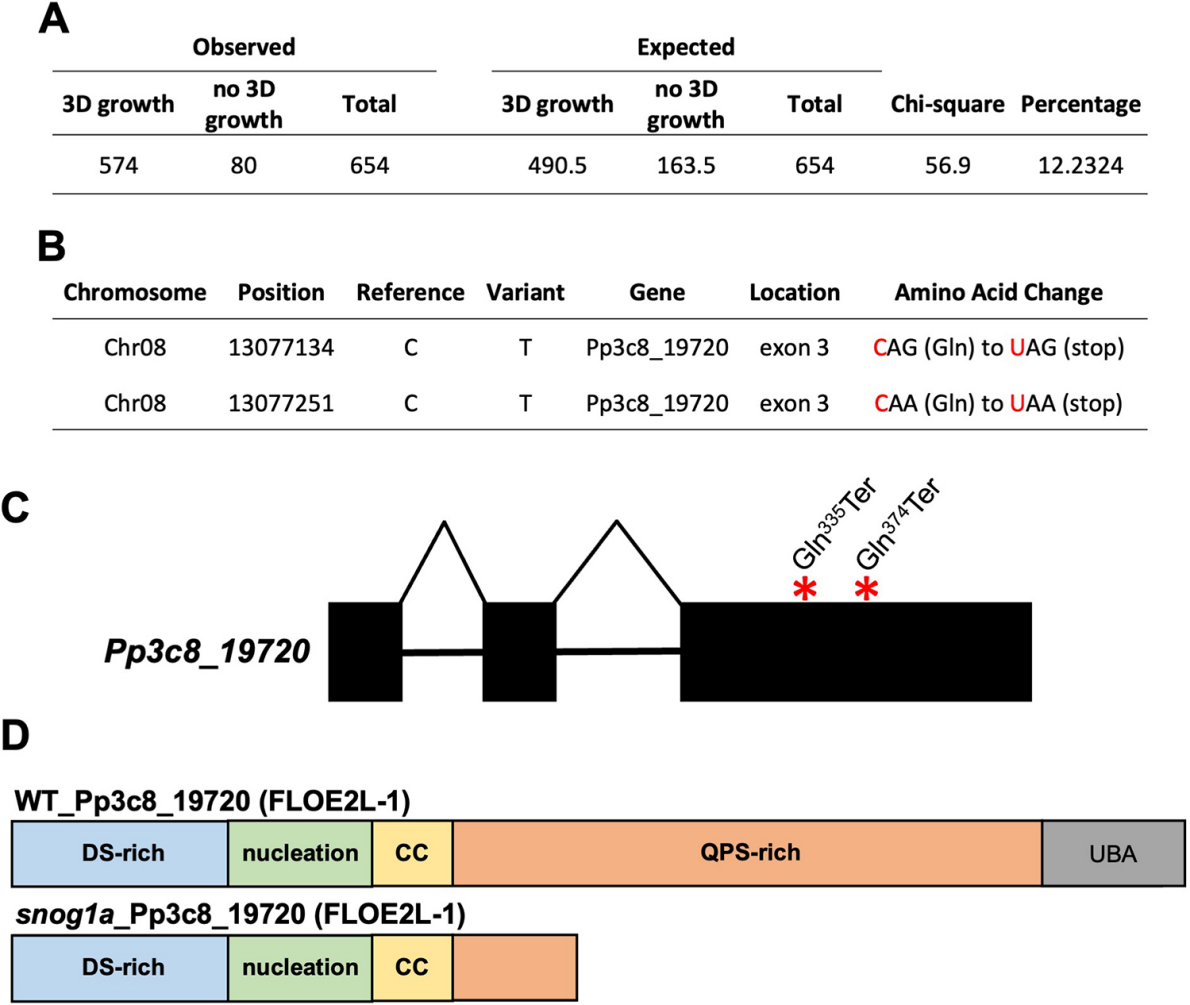
**Identification of the causative mutation in the *snog1a* mutant.** (A) Phenotypic analysis of spore progeny derived from a cross between *snog1a* and the Reute::mCherry wild-type strain. The percentage represents the proportion of individuals that cannot initiate 3D growth and thus have the same phenotype as *nog1dis*. (B) Gene candidates identified following interrogation of the genomic locus on chromosome 8. (C) Gene structure diagram of *Pp3c8_19720* highlighting the presence of two termination codons (asterisks) in the third exon (exons, blocks; introns, horizontal lines). (D) The wild-type Pp3c8_19720 protein (top) and the truncated Pp3c8_19720 protein in *snog1a* (bottom).

To identify the causative mutation in the reproductively viable *snog1a* mutant, we obtained phenotypically segregating populations by performing a cross between the *snog1a* mutant (female parent) and the highly fertile non-mutagenized Reute::mCherry strain (male parent) (Perroud et al., 2020). Outcrossing was confirmed by the detection of mCherry in the resulting sporophytes. Because a conventional cross between two different haploid strains was carried out, and at least two genetic loci were mutated (the mutation within the *NOG1* gene, and the unknown mutation that caused the *snog1a* phenotype), we expected one-quarter of the progeny to exhibit the original *nog1dis* mutant phenotype and the remainder to exhibit varying capacities for 3D growth (Fig. S2A). However, we discovered that the observed frequencies were not consistent with the mutation of a single genetic locus in the *snog1a* mutant (Fig. 4A). We prepared genomic DNA from 80 individuals that exhibited the *nog1dis* phenotype and pooled these in equimolar amounts, and this became the ‘control pool’ (all individuals resembled the non-mutagenized parental line, *nog1dis*). We also prepared genomic DNA from 98 individuals with the capacity for 3D growth, and this became the ‘mutant pool’. These two pools were sequenced at 44× coverage alongside both parental lines; the *nog1dis* mutant (generated in the Villersexel wild-type strain) and the Reute::mCherry line. To identify the genomic region containing the *snog1a* mutation(s), bulk segregant analysis was performed. SNPs that differed between the two parental lines were identified as markers and the frequency of each SNP variant (allele) was mapped across the chromosomes to show the proportion of parental contribution at each region. For chromosomal regions not associated with the phenotypes in the two pools, the expected allele frequency was ∼0.5, showing equal contribution from both parents. The expected *SNOG1A* WT (Reute::mCherry parental origin) allele frequency was 1 in the control pool and 0.33 in the mutant pool (Fig. S2B,C). The allele frequencies for both pools were plotted across the 27 chromosomes of the *P. patens* v3.3 genome assembly. It should be noted that completion of the *P. patens* v6.1 genome assembly has since established that the *P. patens* genome comprises 26 chromosomes (Bi et al., 2024). We determined that the expected mutant allele frequencies were observed on chromosome 8, which provided a region of the genome to interrogate for causative mutations (Fig. S2D,E). Also, the expected allele frequencies for the *NOG1* locus were apparent on chromosome 1, showing that it had segregated as expected in the two pools (Fig. S2D,E). None of the other chromosomes displayed allele frequencies that would indicate there was another locus associated with the *snog1a* mutant phenotype. One possible explanation for the reduced *nog1dis* mutant phenotype frequency observed was that disruption of the *NOG1* gene affects haploid spore germination and/or the viability of young sporelings.

Our analysis revealed that two C>T transitions generated two distinct in-frame termination codons (Gln^335^Ter and Gln^374^Ter) in a single gene (Pp3c8_19720) (Fig. 4B,C). We cloned and sequenced the genomic locus containing these two mutations and confirmed that both point mutations were present in the *snog1a* mutant but absent from both the *nog1dis* mutant and the Reute::mCherry line (Fig. S3). In addition, sequencing of the corresponding transcript confirmed the presence of these point mutations but did not reveal any nonsense-associated altered splicing in the *snog1a* mutant, a phenomenon observed in both previously described *nog1-R* and *nog2-R* mutants (Supplementary Dataset 1) (Moody et al., 2018a, 2021).

The gene mutated in the *snog1a* mutant encodes a protein with a C-terminal UBA, and thus the protein domain architecture resembles that of the NOG1 protein (Moody et al., 2018a). To infer phylogenetic relationships for the putative SNOG1A protein, we set out to retrieve orthologous sequences from the genomes of representatives of the chlorophytes, charophytes, bryophytes, lycophytes, monilophytes, gymnosperms and angiosperms (Supplementary Dataset 2). Remarkably, Pp3c8_19720 shares homology with an *Arabidopsis thaliana* protein (AtFLOE1) that can undergo hydration-dependent phase separation (PS) to regulate seed germination, and related proteins (AtFLOE2 and AtFLOE3) that have been shown to undergo PS in a transient expression system (Dorone et al., 2021). As with the analyses conducted by Dorone et al. (2021), our own phylogenetic analyses revealed the presence of two discrete clades, a FLOE1-like (FLOE1L) and a FLOE2-like (FLOE2L) clade. However, while it was previously stated that the FLOE1L clade was restricted to seed plants, we found FLOE1L representatives in the seed plants in addition to the monilophytes. Conversely, the FLOE2L clade included Pp3c8_19720, as well as three additional *P. patens* paralogues, and homologues in most land plants except for hornworts, where they appear to have been lost. FLOE2L homologues were also identified in some green algal lineages (Fig. S4, Supplementary Dataset 2). Given the significant homology between Pp3c8_19720 and representatives within the FLOE2L clade, we hereafter refer to Pp3c8_19720 as FLOE2L-1.

The AtFLOE1, 2 and 3 proteins contain a predicted folded domain (known as the nucleation domain), a coiled-coil domain (CC), a UBA domain at the C-terminus and two disordered regions; one enriched for aspartic acid and serine (DS-rich domain) and one enriched for glutamine, proline and serine (QPS-rich domain) (Dorone et al., 2021). Alignment of FLOE2L-1 with AtFLOE1, 2 and 3 confirmed that these protein domains were conserved in FLOE2L-1 (Fig. 4D, Fig. S5). As a result of the nonsense mutations introduced into *FLOE2L-1*, a truncated protein was formed in *snog1a*, which was missing most of the QPS-rich domain in addition to the UBA (Fig. 4D). Conversely, we detected the wild-type version of the *FLOE2L-1* transcript in the second *suppressor of nog1* mutant (*snog1b*), suggesting that mutations in a different gene are responsible for the *snog1b* mutant phenotype (Supplementary Dataset 1).

**Fig. 5.**
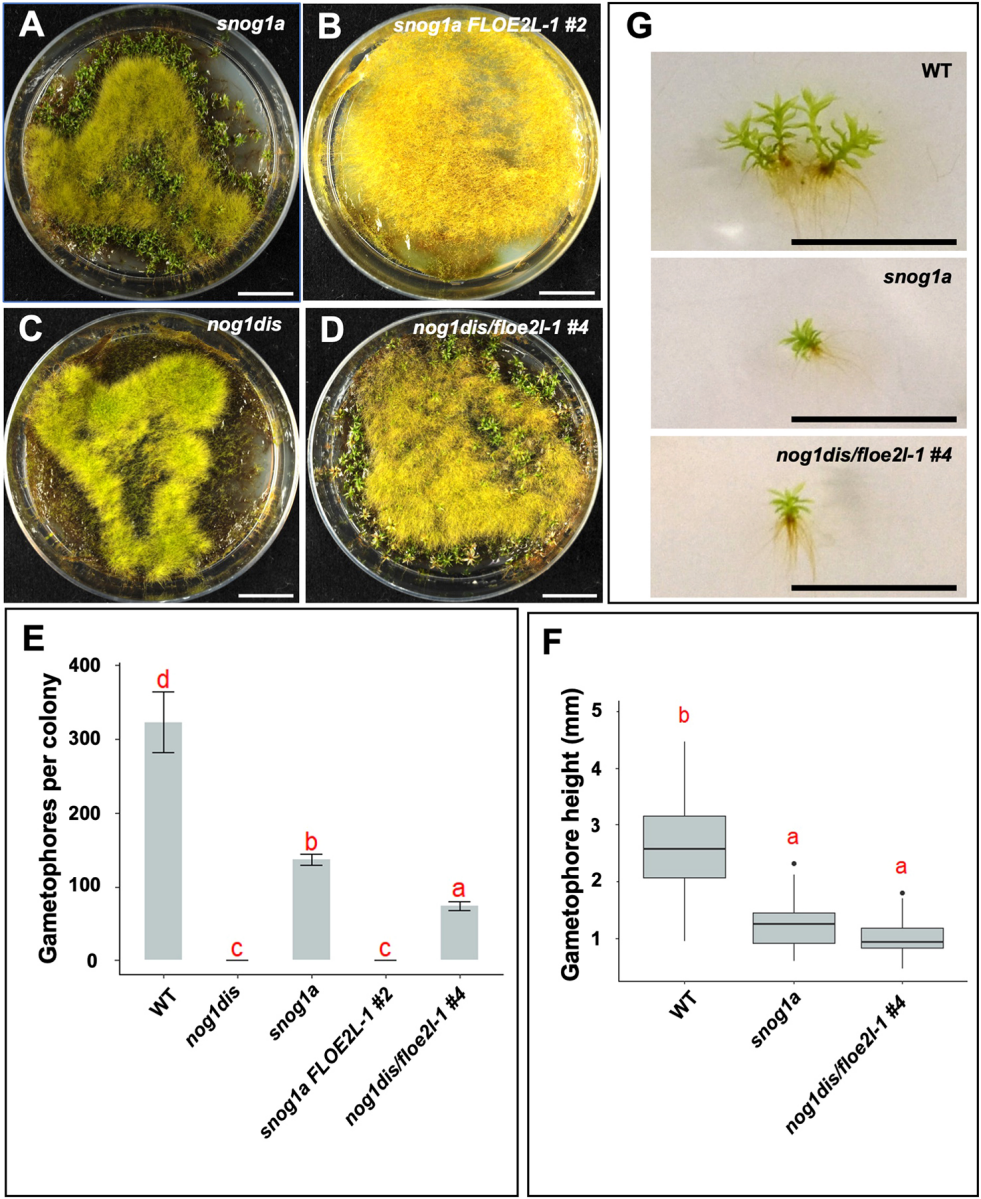
**Confirmation of the causative mutation in the *snog1a* mutant.** (A,B) Representative images of 10-week-old *snog1a* (A) and *snog1a* complemented with the wild-type version of the *FLOE2L-1* transcript (B). (C,D) Representative images of 10-week-old *nog1dis* (C) and the *nog1dis*/*floe2l-1* #4 double disruptant (D). (E) Mean number of gametophores formed per colony in wild type (*n*=3), *nog1dis* (*n*=5), *snog1a* (*n*=5), *snog1a* complemented with wild-type *FLOE2L-1* (*n*=5) and the *nog1dis/floe2L-1#4* double disruptant mutant (*n*=5) ±s.e.m. (F) Mean height of gametophores (mm) from wild type (*n*=135), *snog1a* (*n*=55) and *nog1dis/floe2L-1* #4 (*n*=53). Box plot shows mean values (middle bars) and first to third interquartile ranges (boxes); whiskers indicate 1.5x the interquartile ranges; dots indicate outliers. The red letters in E and F indicate the result of Tukey's HSD post-hoc test – lower case letters are used to label means, such that bars bearing different letters are statistically different from one another with a *P*adj value of <0.05. (G) Representative images of gametophores from wild type, *snog1a* and *nog1dis/floe2L-1* #4. Scale bars: 1 cm.

To confirm that we had correctly identified the causative mutation, we used the rice actin promoter to drive the expression of a wild-type version of *FLOE2L-1* in the *snog1a* mutant. The resulting line exhibited a reversion to the 3D-defective phenotype seen in the *nog1dis* mutant, with a complete absence of gametophores (Fig. 5A,B,E and Fig. S6). We also generated an independent line in which *FLOE2L-1* was disrupted in the *nog1dis* mutant. The construct was designed to replace the endogenous *FLOE2L-1* sequence with a G418 resistance cassette (Fig. S7A). Although appropriate 5′ integration had occurred, it appeared that the *FLOE2L-1* coding sequence had been retained but was likely shifted away from the *FLOE2L-1* gene promoter (Fig. S7B). Regardless, in the *nog1dis*/*floe2l-1* double disruptant mutants, suppression of *FLOE2L-1* expression was evident based on quantitative RT-PCR (Fig. S7C). Furthermore, the phenotype of the original *snog1a* mutant had been successfully recapitulated (Fig. 5C-G). Thus, the causative mutation of *snog1a* resides in a gene that encodes a FLOE-related protein.

### Cuticle biosynthesis is restored in the *snog1a* mutant

As there is a strong correlation between the initiation of 3D growth and cuticle biosynthesis, and it is likely that one is a prerequisite for the other, we performed Toluidine Blue staining to test for cell wall permeability. As anticipated, the wild-type protonema stained intensely while the gametophores remained unstained (Fig. S8A). The same pattern of staining was observed in the *snog1a* mutant, which exhibits partial restoration of 3D growth (Fig. S8B). The *nog1dis* mutant developed only protonemal tissues that stained intensely (Fig. S8C), and this staining pattern was matched in the *snog1a* mutant complemented with a functional copy of *FLOE2L-1* (Fig. S8D). Furthermore, in an independently generated line in which the *snog1a* mutant phenotype had been recapitulated, the formation of a functional cuticle had been restored (Fig. S8E).

### RNA-seq reveals a suppression of the transcriptional response to cytokinin in *nog1dis* and *snog1a*

To further explore the interplay between *NOG1*, *FLOE2L-1* and cytokinin, an RNA-seq experiment was performed using 2-week-old protonemal tissue, which coincides with the onset of the 2D-to-3D growth transition. We set out to identify those genes that were differentially expressed in wild type, *nog1dis* and *snog1a.* We were also keen to identify those genes that were induced or repressed by cytokinin in both wild type and in *snog1a*, but not in the *nog1dis* mutant. This would ultimately reveal the transcriptional changes needed to restore 3D growth to *nog1dis*. Importantly, we were able to again confirm the complete absence of *NOG1* expression in both *nog1dis* and *snog1a* (Fig. S9A, Supplementary Dataset 3). We also exploited our transcriptome data to investigate the expression of all four FLOE2L genes during the 2D-to-3D growth transition. We determined that *FLOE2L-1* was expressed at equivalent levels in both wild type and in *nog1dis*, but was downregulated in the *snog1a* mutant, likely due to nonsense-mediated mRNA decay. We could detect the transcripts associated with both *Pp3c24_13440* (*FLOE2L-2*) and *Pp3c11_23810* (*FLOE2L-3*) genes. Although the expression of *FLOE2L-3* remained unchanged in *snog1a*, the *FLOE2L-2* gene was slightly upregulated compared to wild type and the *nog1dis* mutant (Fig. S9B). We could not detect the *Pp3c7_1641* (*FLOE2L-4*) transcript in our dataset, although we could amplify moderate levels of transcript using RT-PCR (Fig. S9C). Thus, all four FLOE2L genes are expressed at the developmental stage in which 3D growth is being established.

**Fig. 6.**
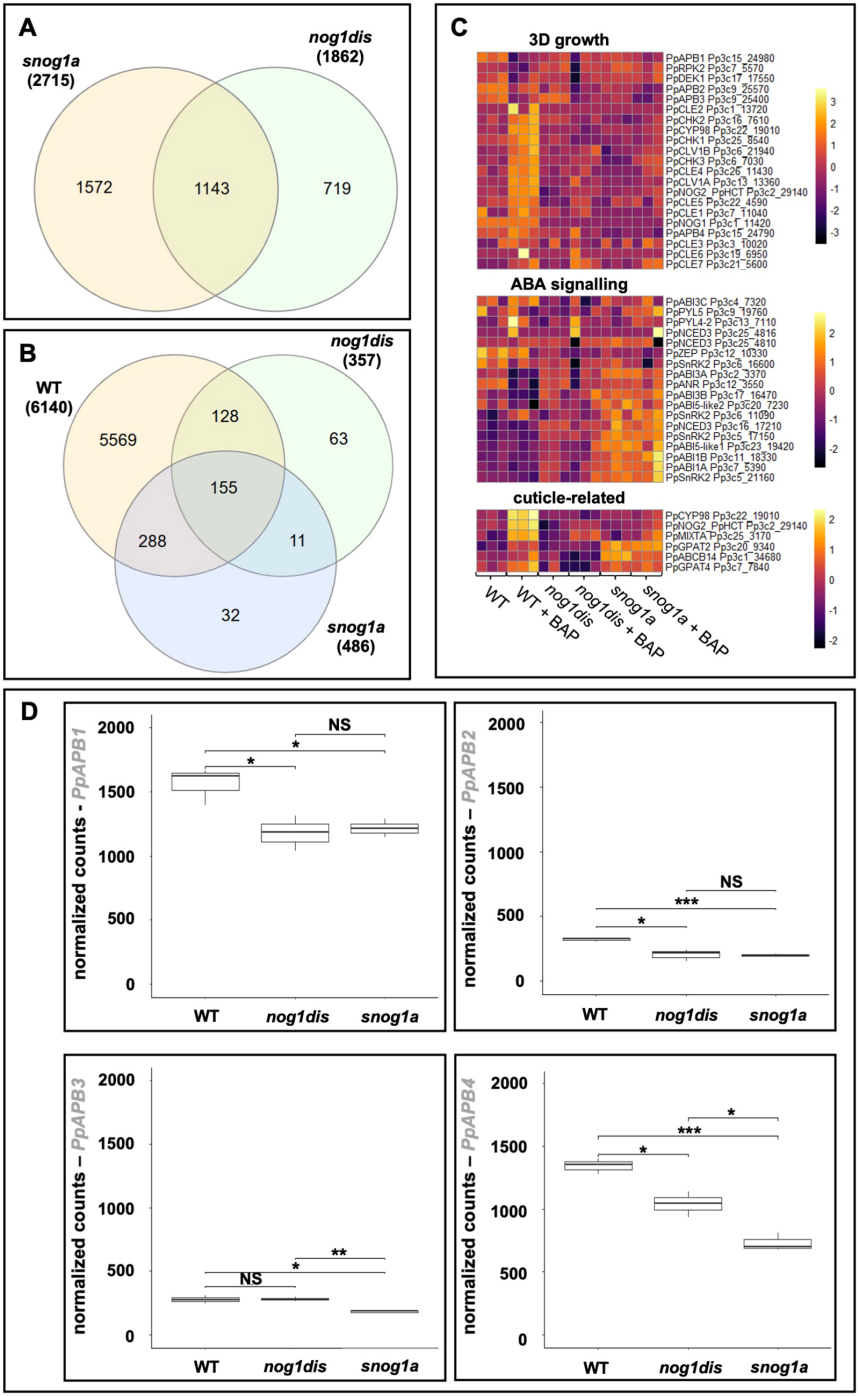
**Transcriptome comparisons of wild type, *nog1dis* and the *snog1a* mutant.** (A) Venn diagram showing the overlap of differentially expressed genes in *nog1dis* and *snog1a* compared to wild type. (B) Venn diagram showing the overlap of differentially expressed genes in response to cytokinin in wild type, *nog1dis* and the *snog1a* mutant. (C) Heat maps showing the normalized counts for each sample, log2 transformed and scaled by row. This visualizes the relative expression levels of selected genes involved in 3D growth (top), ABA signalling (middle) and cuticle biosynthesis (bottom) in *P. patens*. (D) Normalized read counts for *APB1*, *APB2*, *APB3* and *APB4* from RNA-seq data showing relative expression levels in wild type, *nog1dis* and the *snog1a* mutant (Student's *t*-test, two-tailed, unpaired; *P<0.05, **P<0.01, ***P<0.001; NS, not significant).

In the *nog1dis* mutant, 752 genes were upregulated and 1110 genes were downregulated relative to wild type, which collectively comprised 5.80% of all expressed genes identified. In the *snog1a* mutant, 1350 genes were upregulated and 1365 genes were downregulated relative to wild type, which collectively comprised 8.46% of all expressed genes (Fig. S10A). There were 1143 genes differentially expressed in both *nog1dis* and *snog1a* compared to wild type, while there were 719 genes disrupted only in *nog1dis* and 1527 genes affected only in *snog1a* (Fig. 6A). Furthermore, there were significantly fewer genes that were differentially expressed in the *snog1a* mutant relative to *nog1dis*. Notably, we identified a total of 349 genes that were differentially expressed in *nog1dis* compared to wild type, but not in the *snog1a* mutant. This included a considerable number of transcription factors and demonstrated that reactivation of certain transcriptional networks had occurred to facilitate the partial restoration of 3D growth in the *snog1a* mutant (Supplementary Dataset 4).

Gene Set Enrichment Analysis (GSEA) using Gene Ontology (GO) biological process terms was performed to reveal those pathways that had been most impacted. In *nog1dis*, processes associated with the localization and transport of proteins had been activated, and ubiquitin-dependent processes had been suppressed compared to wild type (Fig. S10B). In *snog1a*, ubiquitin-dependent processes were similarly suppressed, and processes related to responses to abiotic and biotic stress or carbohydrate metabolism were upregulated compared to wild type (Fig. S10C). When examined more closely, we discovered that *NOG1* was the only gene associated with the ubiquitin-dependent GO term in both *nog1dis* and in the *snog1a* mutant. When comparing *snog1a* to *nog1dis*, it was evident that hormone signalling pathways, notably ABA signalling, had been activated in the *snog1a* mutant compared to the *nog1dis* mutant (Fig. S10D). When the expression patterns of individual ABA signalling genes were compared, we determined that several of these showed enhanced expression in *snog1a*, compared to wild type and the *nog1dis* mutant. This included *ABI1A*, *ABI1B* and several SnRK2 genes (Fig. 6C). This suggests that FLOE2L-1 can act as a negative regulator of ABA signalling in certain developmental contexts.

Following cytokinin treatment, 3417 genes were upregulated and 2723 genes were downregulated in wild type relative to the untreated wild-type control, which comprised nearly 20% of all genes identified. In contrast, only 1.11% and 1.52% of genes were differentially expressed in *nog1dis* and *snog1a*, respectively (Fig. 6B, Fig. S11A). This demonstrates that the transcriptional response to cytokinin is strongly suppressed in both mutants, even though the *snog1a* mutant exhibits strong phenotypic responsiveness to exogenously applied cytokinin (Fig. 2).

There are 155 genes that are differentially expressed in a similar manner in wild type, *nog1dis* and *snog1a*, but there are also genes that are specifically induced in either *nog1dis* or the *snog1a* mutant (Fig. 6B). Notably, there is an overlap of 288 genes which are responsive to cytokinin in wild type and *snog1a*, but not in the *nog1dis* mutant. These genes include several kinases and putative transcription factors, as well as three cytokinin oxidase enzymes, a gene that encodes a CHASE domain histidine kinase (*CHK3*; a classical cytokinin receptor) and two auxin efflux transporters (Fig. 6B, Supplementary Dataset 5). These transcriptional changes may explain the restoration of the cytokinin response in the *snog1a* mutant. We also performed GSEA on the cytokinin response comparisons. As expected, cytokinin treatment elicited the activation of processes associated with cell growth, cell wall biogenesis and cell wall organisation in wild type. However, processes associated with photosynthesis were suppressed (Fig. S11B). Although processes associated with cell wall biogenesis and organisation were both activated in *nog1dis* and *snog1a* in response to cytokinin (Figs S11C, S10D), processes associated with cell growth were not activated in the *nog1dis* mutant (Fig. S11C). In *nog1dis*, genes associated with protein synthesis were induced by cytokinin, whereas those associated with intracellular signalling were suppressed (Fig. S11C). Notably we discovered that those genes strongly associated with responses to abiotic stress and ABA signalling were induced by cytokinin in *snog1a* but not in wild type or the *nog1dis* mutant. This suggests some level of hypersensitivity to cytokinin, which may impact on ABA signalling (Fig. S11D).

Given that all the cuticle-defective mutants described so far (Renault et al., 2017; Lee et al., 2020; Moody et al., 2021; Kriegshauser et al., 2021; Zhang et al., 2024) have highly pronounced 3D growth defects, and because the cuticle has been restored in the *snog1a* mutant, we decided to examine the expression of cuticle-related genes. We discovered that cuticle-related genes that are normally strongly induced by cytokinin in wild type are not induced by cytokinin in either *nog1dis* or the *snog1a* mutant (Fig. 6C). This includes *CYP98* and *NOG2/HCT*, enzymes that catalyze steps in the phenylpropanoid pathway leading to cuticle biosynthesis in *P. patens*, and the glycerol-3-phosphate acyltransferase (GPAT) genes *GPAT2* and *GPAT4*, which are required for the formation of cutin monomers (Renault et al., 2017; Lee et al., 2020; Moody et al., 2021; Kriegshauser et al., 2021). Notably, we determined that the expression of *GPAT2* was strongly upregulated and *GPAT4* was moderately upregulated in *snog1a* relative to both wild type and in the *nog1dis* mutant in the absence of cytokinin. Furthermore, the expression of the *ABCB14* gene, which encodes an ATP-binding cassette transporter that has been implicated in cuticle deposition, was also upregulated in *snog1a* compared to wild type and *nog1dis* (Fig. 6C) (Zhang et al., 2024). Thus, there is a differential response to cytokinin in wild type, *nog1dis* and the *snog1a* mutant that likely impacts the regulation of cuticle biosynthesis.

### Restoration of 3D growth in the *snog1a* mutant occurs independently of APB genes

Within our transcriptome datasets, we were able to investigate the expression patterns of genes with known roles in 3D growth in *P. patens*. We observed that genes that were cytokinin responsive in wild type tended to be cytokinin unresponsive in both *nog1dis* and *snog1a*, except for *CHK3* (as described above), which has regained partial responsiveness in the *snog1a* mutant (Fig. 6C). We determined that the expression of the APB genes was downregulated in *nog1dis*, as in the *nog1-R* mutant (Moody et al., 2018a) but also discovered that this downregulation had not been reversed in the *snog1a* mutant as anticipated. Notably, *APB4*, which has been shown to be sufficient for gametophore induction, was further suppressed in *snog1a* compared to *nog1dis* (Aoyama et al., 2012) (Fig. 6D). This suggests that gametophore initiation can occur independently of the APB transcription factors, or that their downstream target(s) have been stabilized in the *snog1a* mutant.

## DISCUSSION

The acquisition of apical cells with the capacity for 3D growth occurred in the last common ancestor of land plants, and enabled the diverse morphologies seen across the planet today (Kenrick and Crane, 1997; Delwiche and Cooper, 2015). We previously showed that the *NOG1* gene is required for 3D growth in *P. patens*, an extant representative of the bryophytes. Notably, in mutants lacking *NOG1* function (*nog1-R*), gametophore initial cell formation is significantly reduced, even in the presence of cytokinin. Furthermore, cell division planes are misoriented in emerging gametophores, which subsequently undergo premature developmental arrest. Thus, *NOG1* promotes the cytokinin-mediated transition from 2D to 3D growth and positively regulates the orientation of cell divisions required to establish a tetrahedral apical cell (Moody et al., 2018a).

To reveal new insights into the genetic interaction network underpinning the transition from 2D to 3D growth, we first recapitulated the *nog1-R* mutant phenotype in an independent *nog1* disruption mutant (*nog1dis*), in which *NOG1* was not expressed (Fig. S1). The *nog1dis* mutant was generated using a targeted approach and thus lacked the background SNPs that were introduced into the *nog1-R* mutant by UV-based mutagenesis. We then performed a UV-mediated suppressor screen to identify mutations that alleviated the *nog1dis* mutant phenotype to permit the reversion to 3D growth. The primary focus of this paper was the characterization of the *snog1a* mutant identified in this screen.

Similarly to the original *nog1-R* mutant, the *nog1dis* mutant was unable to form gametophores even in the presence of cytokinin (Figs 1 and 2). Mutants in which all four APB genes have been disrupted exhibit a similar level of cytokinin-unresponsiveness to both *nog1-R* and *nog1dis* mutants and fail to make the 3D growth transition (Aoyama et al., 2012). Furthermore, we previously observed that APB genes are downregulated when *NOG1* is absent (Moody et al., 2018a). Thus, *NOG1*, along with the APB genes are integral regulators of cytokinin perception during the switch from 2D to 3D growth. As the *snog1a* mutant exhibits responsiveness to cytokinin, this suggested that those cytokinin signalling components, ordinarily repressed in *nog1dis*, had been reactivated in the *snog1a* mutant. This demonstrates that the phenotypes observed in both *nog1dis* and *snog1a* can be attributed to alterations in cytokinin perception. Thus, this study has begun to shed light on the potential mechanism underlying cytokinin-mediated 3D growth initiation, a topic that has fascinated biologists for several decades (Brandes and Kende, 1968; Ashton et al., 1979; Reski and Abel, 1985; Schulz et al., 2000, 2001; von Schwartzenberg et al., 2007, 2016; Cammarata et al., 2022).

Although the formation of gametophore initial cells was not fully restored in the *snog1a* mutant, those that formed usually followed a wild-type pattern of development to achieve the establishment of a tetrahedral apical cell (Fig. 3). It has previously been shown that the positioning of cell division planes in emerging gametophores is dependent on microtubules (Kosetsu et al., 2017; Kozgunova et al., 2022). Thus, it is likely that the cell division orientation defects observed in the *nog1dis* mutant are due to aberrant organisation of the microtubule cytoskeleton. Since the correction of division plane orientation observed in the *snog1a* mutant is accompanied by the restoration of a cytokinin response, we speculate that the microtubule organisation that occurs during the 3D growth transition is dependent on cytokinin. This is consistent with other reports in the literature (Montesinos et al., 2020).

There is an increasing volume of literature that describes the regulatory role of cuticle-related genes in the 3D growth transition (Renault et al., 2017; Lee et al., 2020; Kriegshauser et al., 2021; Moody et al., 2021). These mutants invariably exhibit perturbations in the frequencies of gametophore initial cells that form, and those that do form exhibit division orientation defects that arrest gametophore development. Furthermore, the expression of these genes has been shown to be induced by cytokinin (Moody et al., 2021). Although we understand that a cuticle is absent in the protonema but observed in gametophores, the developmental stage at which cuticle biosynthesis initiates remains unclear (Renault et al., 2017; Lee et al., 2020; Kriegshauser et al., 2021). In several of the 3D-defective mutants described in the literature so far, including the *nog1-R* mutant, the cuticle is invariably absent (Aoyama et al., 2012; Renault et al., 2017; Moody et al., 2018a; Kriegshauser et al., 2021; Zhang et al., 2024). Thus, it is possible that the acquisition of the cuticle was a prerequisite for 3D growth, and that the mechanical constraints imposed by the cuticle alter the division properties of cells formed in the steps toward the establishment of a tetrahedral apical cell. To support this hypothesis, the cuticle is notably absent in the *nog1dis* mutant but has been partially restored in the *snog1a* mutant, in which the partial reversion to 3D growth has occurred (Fig. S8).

Our mapping approach and phylogenetic analyses revealed that the gene mutated in the *snog1a* mutant (*Pp3c8_19720*) was a homologue of the previously characterized FLOE genes in *Arabidopsis*; *AtFLOE1*, *2* and *3* (Fig. 4, Figs S3 and S4) (Dorone et al., 2021). Dorone et al. have demonstrated that AtFLOE1 undergoes reversible hydration-dependent liquid-liquid phase separation (LLPS) in the embryo, and that this process is dependent on the presence of a QPS-rich disordered domain. LLPS drives the formation of AtFLOE1 condensates when a seed is hydrated, but AtFLOE1 remains dispersed in desiccated seeds. Since loss of AtFLOE1 function can promote germination during drought or salt stress, it is thought that AtFLOE1 functions to inhibit seed germination in unfavourable conditions. The authors also demonstrated that AtFLOE2 and AtFLOE3, in addition to representatives from algae and bryophytes, undergo LLPS in a transient expression system (Dorone et al., 2021).

AtFLOE1 is a member of the FLOE1L clade, which contains only those representatives from the monilophytes and seed plants. On the other hand, AtFLOE2 and AtFLOE3 reside within the FLOE2L clade, which contains representatives from algal species as well as all land plant lineages. This includes *Pp3c8_19720* in addition to three additional homologues in *P. patens*. We were able to complement the *snog1a* mutant phenotype with a full-length version of the wild-type coding sequence and recapitulate the phenotype by generating an independent double disruptant mutant (Fig. 5). The confirmation of gene identity enabled us to amend the name of *Pp3c8_19720* to *FLOE2L-1*. The presence of three additional FLOE2L homologues in the *P. patens* genome suggests that these genes may function redundantly. Thus, we hypothesize that higher order mutants of these genes will exhibit a progressive restoration of 3D growth, and indeed cuticle formation. FLOE2L genes were notably absent from hornworts, which suggests that the genetic toolkit underpinning 3D growth processes in hornworts is somewhat distinct.

Our RNA-seq experiments have revealed that transcriptional responses to cytokinin are mostly suppressed in both *nog1dis* and *snog1a*. Although there are a few genes that have regained cytokinin responsiveness in *snog1a*, it is not yet clear how this results in a cytokinin-responsive phenotype that is like that seen in wild type. Notably, ABA signalling processes were activated in the *snog1a* mutant. ABA signalling is an important regulator of drought stress responses across all land plants and also acts as an inhibitor of germination in seed plants (Kermode, 2005; Wang et al., 2010). It has been shown that AtFLOE1 responds to water availability in the *Arabidopsis* seed and acts to repress germination if water is scarce. However, the mechanism by which AtFLOE1 represses germination is not known (Dorone et al., 2021). If there is functional conservation in FLOE function across land plants, then an interaction between FLOE genes and the ABA signalling pathway may hint at this mechanism.

LLPS is a phenomenon that has been increasingly linked to important developmental processes in plants (Fang et al., 2019; Huang et al., 2021; Cao et al., 2023). Furthermore, there have been reports that LLPS enables ubiquitin-binding shuttle proteins (i.e. those containing UBAs) to degrade ubiquitinated substrates (Dao and Castañeda, 2020). Similar to the three other FLOE2L genes identified in *P. patens*, *FLOE2L-1* encodes a protein that contains: a disordered QPS-rich domain, previously shown to serve as a prerequisite for LLPS; CC and nucleation domains; a disordered DS-rich domain; and a UBA. The presence of the UBA suggests that FLOE2L-1 plays a role in protein degradation, which is curiously reminiscent of the NOG1 protein. Due to the presence of two in-frame premature stop codons in the *FLOE2L-1* transcript, the *snog1a* mutant lacks most of the QPS-rich disordered domain as well as the UBA (Fig. 4). Thus, we hypothesize that FLOE2L-1 undergoes cell-type dependent LLPS to compartmentalize the cellular components required to degrade a ubiquitinated repressor of the 2D to 3D growth transition. In support of this notion, it has been reported that E3 ligases involved in protein degradation processes are able to function within condensates (Dao and Castañeda, 2020). Thus, we propose LLPS as a mechanism by which the induction of 3D growth can be rapidly triggered in response to both intrinsic and extrinsic cues, at the correct stage of development.

### Conclusion

**Fig. 7.**
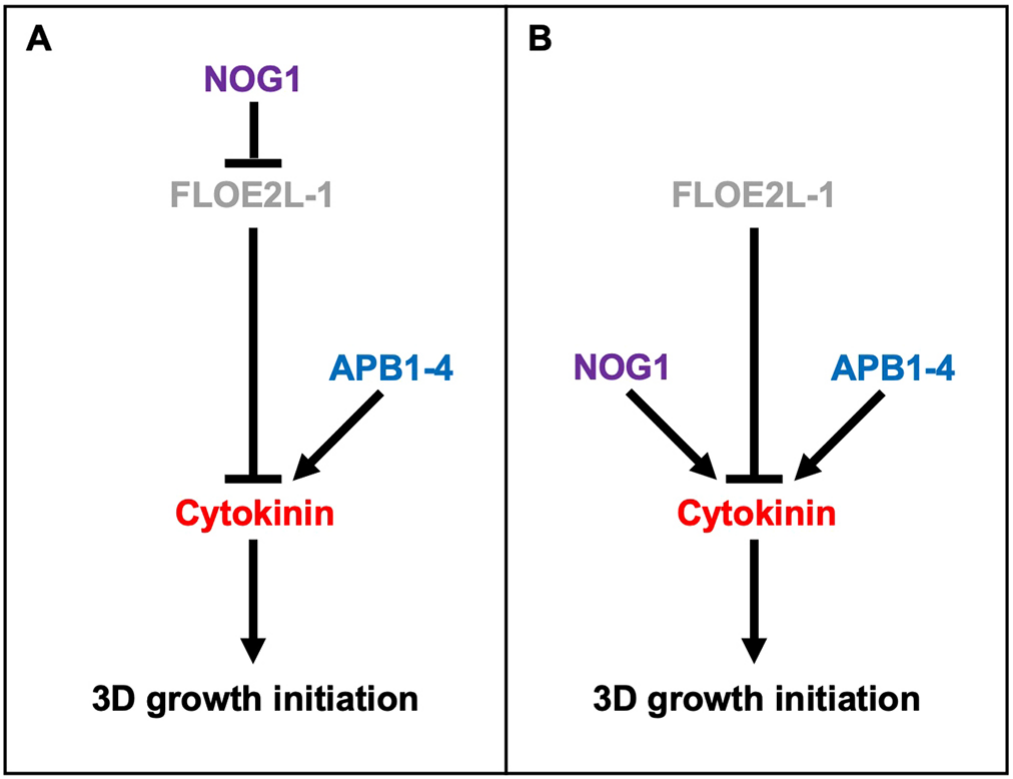
**Speculative model for 3D growth regulation in *P. patens*.** (A,B) Two alternative speculative models highlighting the possible relationships between NOG1, FLOE2L-1 and the APB transcription factors.

Since the loss of FLOE2L-1 function can reverse the 3D-defective phenotype of the *nog1dis* mutant phenotype, we have demonstrated that FLOE2L-1 acts as a negative regulator of 3D growth. We have also shown that the expression of the APB genes is not corrected in the *snog1a* mutant, even though the capacity for 3D growth has been recovered. Thus, we have devised two alternative models for NOG1 function (Fig. 7). The first model proposes that NOG1 acts upstream of, and represses the activity of FLOE2L-1, which in turn induces the degradation of protein(s) that repress cytokinin-mediated 3D growth initiation independently of the APB transcription factors (Fig. 7A). Alternatively, the second model proposes that NOG1 and FLOE2L-1 act antagonistically to regulate the degradation of protein(s) that mediate the respective promotion or repression of cytokinin-mediated 3D growth initiation (Fig. 7B).

## MATERIALS AND METHODS

### *P. patens* growth conditions

To encourage bud development, *P. patens* was grown on cellophane-overlaid BCD medium [1 mM MgSO_4_, 1.84 mM KH_2_PO_4_ (pH 6.5), 10 mM KNO_3_, 45 µM FeSO_4_, 1 mM CaCl_2_, 0.1% Trace Elements Solution: 116 µM AlK(SO_4_)_2_, 220 µM CuSO_4_, 10 mM H_3_BO_4_, 235 µM KBr, 660 µM LiCl, 230 µM CoCl_2_, 190 µM ZnSO_4_, 2 mM MnCl_2_, 170 µM KI, 124 µM SnCl_2_] containing 0.8% agar. For routine propagation, and to stimulate filamentous growth, *P. patens* was grown on BCD medium supplemented with 5 mM ammonium tartrate (BCDAT). To enable propagation, tissues were harvested and homogenized in sterile water using an IKA T-25 digital ULTRA-TURRAX^®^. Tissues were then pipetted onto cellophane-overlaid BCDAT plates in a laminar flow hood. Plates were then placed in a growth cabinet at 24°C with a 16 h light/8 h dark (300 µmol m^−2^ s^−1^) cycle. Protoplasts were regenerated on cellophane-overlaid Protoplast Regeneration Medium [PRMB; BCDAT supplemented with 10 mM CaCl_2_, 0.5% glucose and 6% (w/v) D-mannitol] (Cove et al., 2009).

### Generation of the *nog1dis* mutant

A genomic DNA fragment from −827 bp upstream of the start codon, up to but excluding the start codon of the *NOG1* sequence, was PCR-amplified using NOG1.5FKpnI and NOG1.5RXhoI primers (Table S1) and ligated into KpnI/XhoI cut pAHG1 (a kind gift from Yasuko Kamisugi and Andrew Cuming, University of Leeds, UK) to create pAHG1-*NOG1*-5′. A *NOG1* genomic DNA fragment including the stop codon up to 1420 bp downstream of the *NOG1* stop codon was PCR-amplified using NOG1.3FNotI and NOG1.3RNotI primers and ligated into NotI cut pAHG1-*NOG1*-5′ to create *pNOG1*delH. *pNOG1*delH was linearized using KpnI before transformation into protoplasts isolated from the Villersexel wild-type strain. Stable transformants were selected using 15 mg ml^−1^ Hygromycin B (Sigma-Aldrich, H9773).

### RT-PCR to detect absence of *NOG1* in the *nog1dis* mutant

Total RNA was isolated from 2-week-old Villersexel wild type and the *nog1dis* mutant using the RNeasy kit (Qiagen) according to the manufacturer's instructions. DNase treatment was carried out using TURBO DNase (Ambion) and cDNA synthesis performed using Superscript™ III (Thermo Fisher Scientific), as specified by the manufacturers. The *tubulin* transcript was amplified using tubF and tubR, and the *NOG1* transcript was amplified using either NOG1_GSP.F and NOG1_GSP.R, or NOG1_exon3F and NOG1_exon5R (Table S1).

### RT-PCR to detect *FLOE2L-4* in WT tissues

RNA was extracted from 2-week-old wild-type tissues and converted into cDNA as described above. The *tubulin* transcript was amplified using tubF and tubR, and a portion of the *FLOE2L-4* transcript was amplified using *Pp3c7_1641*_qPCR_F and *Pp3c7_1641*_qPCR_R (Table S1). The amplicon was sanger sequenced by Source Bioscience with the same primers to confirm that the amplicon belonged to the *Pp3c7_1641* transcript.

#### Transcriptome analysis

*P. patens* tissues were initially grown on BCD for 1 week and then transferred to plates containing BCD+1 µM 6-benzylaminopurine (BAP) for another week. Control samples that were not treated with BAP were transferred to a fresh BCD plate containing no BAP at the same stage. For each line and treatment there were three biological replicates. Tissues were harvested and frozen in liquid nitrogen, and then ground with a pestle. Total RNA was then extracted and DNase treated as described above. The quality and quantity of RNA was determined using a NanoDrop™ spectrophotometer. Quality control and sequencing was performed by Novogene (NovaSeq PE150 platform, 150 bp paired end reads). Fastp v0.20.1 was used for quality filtering of the raw reads, adaptor trimming, base correction and over-representation analysis (Chen et al., 2018). Filtered reads were mapped to the reference transcriptome (Phypa_V3), which was obtained from Ensembl Plants (Lang et al., 2018). Mapping and quantification were performed using Salmon v1.5.1 (Patro et al., 2017). The R packages biomaRt v2.46.3 and GenomicFeatures were used to create the TxDb (Durinck et al., 2009; Lawrence et al., 2013), which was used to create a Tx2gene data frame. This was then converted to a matrix using tximport (Soneson et al., 2015). Differentially expressed gene (DEG) analysis was carried out using DeSeq2 v1.30.1 (Love et al., 2014). The log_2_ fold change was shrunk (normal shrinkage type) to adjust for genes expressed at a low level. Statistical comparisons were performed using the Wald test with a false discovery rate (FDR) <0.05. GSEA was carried out using clusterProfiler 4.10.0 (Wu et al., 2021).

#### Quantitative RT-PCR

Quantitative RT-PCR (RT-qPCR) was used to quantify relative transcript levels. There were three biological replicates for each *P. patens* line and three technical replicates for each cDNA sample and water controls. On a 96-well PCR plate, each well contained 2 µl of cDNA template, with 6 µl SYBR^®^ Green PCR Master Mix (Applied Biosystems™), 0.5 µl each of the forward and reverse qPCR primers (5 µM) for the target or control genes (Table S1) and 1 µl water. A StepOnePlus™ Real-Time PCR System and the accompanying StepOne™ Software (Applied Biosystems™), with a relative standard curve quantitation was used to carry out the RT-qPCR. The cycling conditions were 5 min at 95°C, followed by 40 cycles of 15 s at 95°C and 1 min at 60°C. The raw amplification data was processed with Real-time PCR Miner (Zhao and Fernald, 2005), which calculated cycle threshold (CT) values. From the CT values, the expression levels of genes of interest were calculated relative to the control gene using the formula 2^−ΔΔCT^ as in Moody et al. (2018a). An ANOVA test was used to determine if there was statistically significant variation in relative transcript levels between *P. patens* lines.

### UV mutagenesis and screening

One-week old protonemata from the *nog1dis* mutant were digested for 1 h in 1% Driselase dissolved in 8% mannitol. The resulting cell suspension was passed through a 40 µm cell strainer and centrifuged for 3 min at 120 ***g*** without braking. Protoplasts were subsequently washed twice in 8% mannitol, with repeated centrifugation steps in between washes. Cells were resuspended in 6 ml 8% mannitol, counted using a haemocytometer, and then plated at a density of 50,000 cells per plate onto cellophane-overlaid PRMB. Protoplasts were immediately exposed to a 75,000 mJ dose of UV light using a Stratalinker UV Crosslinker and this was performed with the Petri dish lids removed. Following irradiation, the lids were quickly replaced, and the plates were wrapped with micropore tape. Plates were incubated at 24°C in the dark for 24 h, to prevent photoactivatable DNA damage repair, before being transferred to standard growth conditions for a further 2-3 weeks. When visible, regenerating protoplasts were transferred into individual wells of 24-well plates containing BCD medium. After ∼1 month of growth in standard conditions, the mutagenized plants were screened for reversion of the *nog1dis* phenotype (i.e. restoration of gametophore development).

### Imaging

To stain cell walls, tissues were submerged in Propidium Iodide (10 µg ml^−1^) for ∼10 min and then mounted on slides in water. Images were acquired using a Leica SP5 scanning confocal microscope with a 40× water immersive lens. A 488 nm laser was used to excite the Propidium Iodide with 30% laser power and fluorescence was detected at 600-630 nm.

Toluidine Blue staining was performed by submerging tissues in 0.05% (w/v) Toluidine Blue solution (diluted in water) for 30 s to 1 min. The stain was then washed off by rinsing the tissues several times with water until the water remained clear. Images were acquired using a Motic SMZ-171-TLED trinocular stereo microscope fitted with a Chromyx 4K Pro Full HD microscope camera.

### Plant phenotyping

To assay the number of gametophores produced by different *P. patens* lines, tissues were grown on cellophane-overlaid BCDAT plates for 1 week and then the tissues were harvested into separate 50 ml tubes containing 10 ml sterile water. The tissues were each homogenized with an IKA disperser for 20 s, and then 1 ml of homogenate was removed to measure optical density (OD600) in a NanoDrop™ spectrophotometer. The density of the homogenate was normalized by adding the required amount of water to each tube. Then 50 µl of homogenate was pipetted onto BCD medium in small Petri dishes, with five repeats for each line. These plates were grown for 2 months under standard conditions, so that mature gametophores had sufficient time to develop. The number of gametophores formed on each plate was counted. To measure gametophore height, a representative and intact sample from each line was extracted from the tissue, placed in a Petri dish and photographed. The heights were measured in ImageJ by drawing a line from the apex to the base. To determine the cytokinin responsiveness of each line, tissues were grown on BCD medium for 1 week and then the cellophanes were cut and transferred to BCD medium containing 1 µM BAP (or control medium) for 3 days before being examined and images captured using a Leica M165C stereomicroscope equipped with a QImaging Micropublishing 5.1 RTV camera.

### Plant crossing and bulk segregant analysis

Both the *snog1a* mutant and the Reute::mCherry line (Perroud et al., 2019) were grown in magenta pots on BCD medium in standard growth conditions for 2-3 months. Pots were subsequently transferred to sporophyte induction conditions (16 h dark/8 h light, 16°C). After ∼3 weeks, gametophores were examined for the presence of gametangia (archegonia and antheridia). When gametangia were present, the Reute::mCherry line was submerged under water to make a sperm suspension, which was subsequently added to the *snog1a* mutant to allow outcrossing. After ∼4-6 weeks, the resulting sporangia were removed carefully using forceps and examined under a Leica M165C fluorescence stereomicroscope to detect mCherry expression. This demonstrated a successful outcrossing event. Sporangia were sterilized in 70% ethanol for 4 min, washed three times with sterile water and then incubated at 4°C in the dark for 1 week. In a laminar flow hood, the sporangia were transferred to a 15 ml tube containing 10 ml of sterile water and then the sporangia ruptured with a pipette tip. Then 1 ml of spore suspension was plated onto each of ten cellophane-overlaid BCDAT plates and then grown in standard conditions to permit germination and subsequent growth. Individual sporelings were then transferred to wells of 24-well plates containing BCD medium and grown for a further 4-6 weeks. The progeny were screened for the presence or absence of gametophores and sorted into two populations: *nog1dis*-like (control pool, 80 individuals) and *snog1a*-like (mutant pool, 98 individuals). Genomic DNA was then extracted from 1-week-old protonemal tissues prepared from each individual line.

### Isolation of genomic DNA

Immediately before use, 0.07% (v/v) 2-mercaptoethanol and 0.1% (w/v) ascorbic acid were added to aliquots of extraction buffer [100 mM Tris-HCl (pH 8.0), 1.42 M NaCl, 2% CTAB, 20 mM EDTA, 2% PVP-40]. The aliquots of extraction buffer were pre-warmed at 65°C in a water bath. One plate of 1-week-old protonemal tissues, grown on cellophane-overlaid BCDAT plates, was harvested and then blotted on filter paper to remove excess water. Tissues were then placed in a 2 ml microcentrifuge tube and frozen in liquid nitrogen. A miniature pestle was used to grind the tissue into powder, and then 500 µl of pre-warmed extraction buffer was added to the powder while frozen and the mixture was homogenized. An additional 200 µl of extraction buffer was added to the tube, along with 7 µl 10 mg ml^−1^ RNase A, and then incubated at 65°C for 10 min. Then 600 µl of chloroform-isoamyl alcohol (24:1) was added and the tube contents was mixed and centrifuged at 16,000 ***g*** for 10 min. Then the upper aqueous phase of the mixture was transferred to a fresh tube, 0.7 volumes of isopropanol were added, the contents of the tube mixed and spun again immediately at 16,000 ***g*** for 10 min. The pellet was washed in 70% ethanol, allowed to air-dry, and then resuspended in 30 µl nuclease-free water. A NanoDrop™ spectrophotometer was used to assess the quantity and quality of the extracted DNA.

### Preparation of genomic DNA samples for whole genome sequencing

In total, four genomic DNA samples were prepared for sequencing; both parental lines (*nog1dis* and Reute::mCherry) and two pooled samples (a *snog1a*-like ‘mutant pool’ and a *nog1*-like ‘control pool’). The pooled samples were prepared by pooling 1 µg genomic DNA extracted from all individuals in that population (80 individuals in the ‘WT pool’ and 98 individuals in the ‘mutant pool’).

### Candidate identification through bulk segregant analysis

Whole genome sequencing was performed using a NovoSeq X Plus sequencing platform (150 bp PE read lengths, 44× coverage) at Novogene. Bioinformatic analysis was performed as described previously (Moody et al., 2018a, 2021). The presence of the premature termination codons in *Pp3c8_19720* was confirmed by sequencing PCR products, amplified from both wild-type and snog1a-derived genomic DNA, using the primers *Pp3c8_19720*_int_F and *Pp3c8_19720*_int_R (Table S1).

### *P. patens* transformation

Before the transformation, large quantities of plasmid DNA were acquired by midiprep using a QIAGEN plasmid midi kit. Approximately 20 µg of the plasmid was linearized by restriction digest overnight, treated with CIAP and precipitated with sodium acetate. Before starting the transformation, all solutions required were made fresh and filter sterilized using a 0.22 μm syringe filter. The transformation was carried out in a biological safety cabinet. We melted 2 g of polyethylene glycol (PEG) 6000 in a flat-bottomed vial, that had been sterilized in the autoclave, in the microwave for ∼1 min. Then 5 ml mannitol/Ca(NO_3_)_2_ solution [0.8% mannitol, 0.1 M Ca(NO_3_)_2_, 10 mM Tris (pH 8.0)] was added to the molten PEG 6000, mixed and allowed to cool for 2-3 h.

For each *P. patens* line that was to be transformed, 1-2 plates of tissue were harvested. We dissolved 0.1 g Driselase enzyme in 10 ml 8% mannitol for each digest. The tube containing the enzyme solution was wrapped in foil and rocked for ∼10 min at room temperature. The tube was spun for 3 min in a centrifuge at 3300 ***g***, and the supernatant was filter sterilized. *P. patens* tissue was placed in the tube with the Driselase solution, wrapped in foil and rocked very gently until the tissue appeared to be well digested (at least 40 min). The digested tissue was put through a 70 µm cell strainer to separate protoplasts from remaining debris. The protoplasts were spun at no more than 120 ***g***, the supernatant was removed and the protoplasts were resuspended in 6 ml mannitol. This wash was repeated twice more. The cell density of the protoplasts was determined using a haemocytometer. The protoplasts were spun down again and resuspended in MMM [0.5 M mannitol, 0.15 M MgCl_2_, 0.1% MES (pH 5.6)], to achieve a cell density of 1.5×10^6^ cells ml^−1^. The next steps were carried out without delay as the protoplasts cannot tolerate the MMM solution for very long. At least 10 µg of the linearized construct was pipetted into the bottom of a round-bottomed Falcon tube. To this was added 300 µl of protoplast suspension, and 300 µl of the PEG solution was added in drops and the tube was swirled gently after the addition of each drop. The tube was heat-shocked at 45°C in a water bath for 5 min and then incubated at room temperature for a further 5 min. Next, 300 µl 8% mannitol was added to the tube, five times at 3 min intervals and the mixture was gently swirled after each addition. Then, 1 ml 8% mannitol was added to the tube five times at 3 min intervals, gently tilting the tube after each addition to mix it. The tube was centrifuged at 120 ***g***, supernatant removed, and the cells were resuspended in 3 ml 8% mannitol. This suspension was gently pipetted onto three PRMB plates overlaid with cellophanes (1 ml for each plate). These plates were sealed with micropore tape and wrapped in foil for 24 h. Subsequently, they were unwrapped and placed in normal growing conditions for 5-7 days. Then the cellophanes, with the regenerating protoplasts, were transferred to selective BCDAT plates containing the appropriate selective agent. After another week on selective plates, the cellophanes were transferred to BCDAT plates with no selective agent to allow recovery. After another 1-2 weeks, the surviving *P. patens* colonies were individually transferred to selection BCD plates, to select for stably transformed colonies. Following another 1-2 weeks on selection plates, colonies that survived both rounds of selection were transferred to non-selective BCD plates and allowed to grow and generate tissue for further study.

### Generation of *snog1a* complementation lines

RNA was extracted from 2-week-old wild-type tissue using an RNeasy kit (Qiagen) and treated with Turbo DNase (Ambion), according to the manufacturer's specifications. cDNA was then synthesized using Superscript™ III Reverse Transcriptase (Thermo Fisher Scientific). Subsequently, the *Pp3c8_19720* transcript was PCR-amplified (excluding the stop codon) using *Pp3c8_19720*.FSalI and *Pp3c8_19720*.R_NOSTOP_HindIII and ligated into pZAG1 to create Act1p::*Pp3c8_19720*-GFP. This construct was linearized with SacII for subsequent transformation into the *snog1a* mutant. Stable transformants were selected using 100 µg ml^−1^ Zeocin (Invitrogen, R25001). Genotyping was performed using the primers putativeSNOG1A_genomic2_F and putativeSNOG1A_genomic2_R (Table S1, Fig. S6).

### Generation of a *snog1a* deletion mutant

A deletion construct was designed. The construct consisted of the FLOE2L-1 5′ flanking sequence, with a small section of the FLOE2L-1 CDS and a FLOE2L-1 3′ flanking sequence. The 5′ and 3′ sequences were inserted either side of a G418 resistance cassette. The construct was synthesized by TWIST Bioscience. The product was verified by Sanger sequencing. Before transformation into protoplasts isolated from the *nog1dis* mutant, the plasmid was linearized with PvuI. Stable transformants were selected using 40 µg ml^−1^ G418. 5′ integration of the deletion construct was confirmed using the primers snog1a_del_genotyping_F and snog1a_del_genotyping_R (Table S1, Fig. S7).

### Phylogenetics

For each of the species included in the analysis, proteome sequences (primary transcript only) were obtained. The data used were: *Physcomitrium patens* v3.3 (Lang et al., 2018), *Marchantia polymorpha* v3.1 (Bowman et al., 2017), *Selaginella moellendorffii* v1.0 (Banks et al., 2011), *Oryza sativa* v7.0 (Ouyang et al., 2007), *Zea mays* PH207 v1.1 (Hirsch et al., 2016), *Sorghum bicolor* v3.1.1 (McCormick et al., 2018), *Brachypodium distachyon* v3.1 (Vogel et al., 2010), *Arabidopsis thaliana* Araport11 (Cheng et al., 2017), *Solanum lycopersicum* ITAG2.4 (Tomato Genome Consortium, 2012), *Medicago truncatula* Mt4.0v1 (Tang et al., 2014), *Populus trichocarpa* v3.1 (Denoeud et al., 2014), *Micromonas pusilla* CCMP1545 v3.0 (Worden et al., 2009), *Ostreococcus lucimarinus* v2.0 (Palenik et al., 2007), *Chara braunii* S276v1.0 (Nishiyama et al., 2018), *Chlamydomonas reinhardtii* v5.5 (Merchant et al., 2007), *Amborella trichopoda* v1.0 (Amborella Genome Project, 2013), *Botryococcus braunii* v2.1 (Browne et al., 2017), *Anthoceros agrestis* Oxford (Li et al., 2020), *Azolla filiculoides* v1.1 (Li et al., 2018), *Brassica rapa* FPsc v1.3 (DOE-JGI, http://phytozome.jgi.doe.gov/), *Ceratodon purpureus* R40 v1.1 (Carey et al., 2021), *Ceratopteris richardii* v2.1 (Marchant et al., 2021), *Chlorokybus atmophyticus* CCAC 0220 v1.1 (Wang et al., 2020), *Klebsormidium nitens* NIES-2285 v1.1 (Hori et al., 2014), *Mesostigma viride* NIES-296 (Liang et al., 2020), *Spirogloea muscicola* CCAC 0214 (Cheng et al., 2019), *Mesotaenium endlicherianum SAG 12.97* (Cheng et al., 2019), *Coccomyxa subellipsoidea* C-169 v2.0 (Blanc et al., 2012), *Dunaliella salina* v1.0 (Polle et al., 2017), *Volvox carteri* v2.1 (Prochnik et al., 2010), *Porphyra umbilicalis* v1.5 (Brawley et al., 2017), *Sphagnum fallax* v1.1 (Healey et al., 2023), *Diphasiastrum complanatum* v3.1 (DOE-JGI, http://phytozome-next.jgi.doe.gov/), *Gingko biloba* v2021 (Liu et al., 2021), *Glycine max* Wm82 ISU-01 v2.1 (DOE-JGI, http://phytozome.jgi.doe.gov), *Gossypium raimondii* v2.1 (Paterson et al., 2012), *Musa acuminata* v1 (D'Hont et al., 2012), *Panicum hallii* v3.2 (Lovell et al., 2018), *Salvinia cucullata* v1.2 (Li et al., 2018), *Spirodela polyrhiza* v2 (Wang et al., 2014), *Thuja plicata* v3.1 (Shalev et al., 2022), *Vitis vinifera* v2.1 (Jaillon et al., 2007), *Saccharomyces cerevisiae* R64-1-1 (Liachko et al., 2013), *Drosophila melanogaster* BDGP6.32 (Adams et al., 2000) and *Homo sapiens* GRCh38 (Lander et al., 2001).

With this set of proteomes, OrthoFinder was used to identify orthogroups (Emms and Kelly, 2019). The orthogroup containing *Pp3c8_19720* was selected and the protein sequences were aligned using MAFFT (L-IN-SI method) and a gene tree constructed using IQ-TREE, with automatic model selection (ModelFinder) and ultrafast bootstrapping (UFBoot) with 1000 replicates (Hoang et al., 2018; Kalyaanamoorthy et al., 2017; Katoh and Standley, 2013; Nguyen et al., 2015). Tree was rooted and edited in Interactive Tree of Life (iTOL) (Letunic and Bork, 2016).

## Supplementary Material

10.1242/develop.204508_sup1 Supplementary information
