# Supplementary material for: A FLOE-related protein regulates the two-dimensional to three-dimensional growth transition in the moss Physcomitrium patens
Source: Development. 2025 Aug 26;152(16):dev204508. doi: 10.1242/dev.204508 (PMC12448315; doi:10.1242/dev.204508)
Supplement: Supplementary information [file develop-152-204508-s1.pdf]

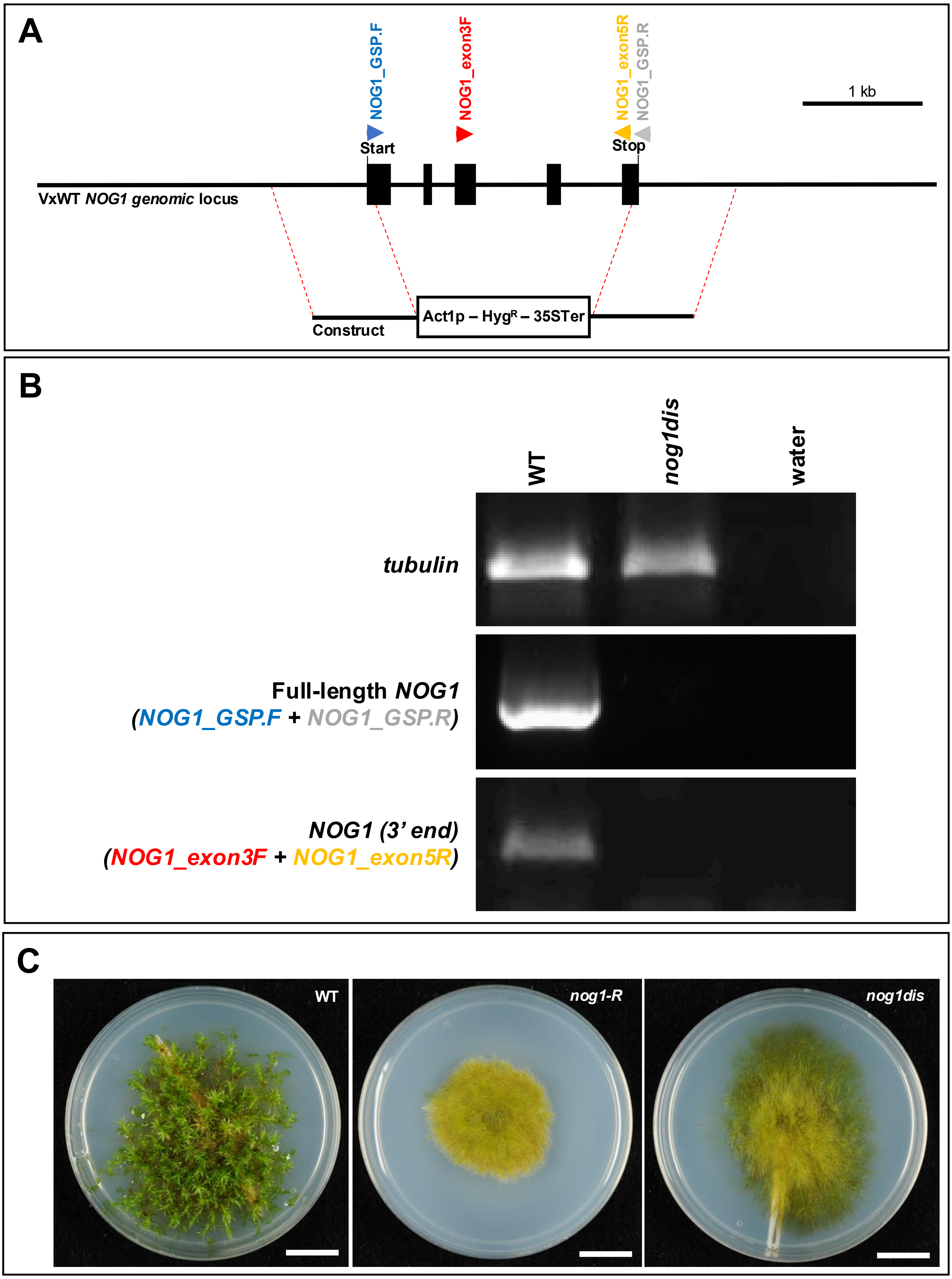
A
NOG1_GSP.F
NOG1_exon3F
NOG1_exon5R
NOG1_GSP.R
1 kb
Start
Stop
VxWT NOG1 genomic locus
Act1p – HygR – 35STer
Construct
B
WT
nog1dis
water
tubulin
Full-length NOG1
(NOG1_GSP.F + NOG1_GSP.R)
NOG1 (3' end)
(NOG1_exon3F + NOG1_exon5R)
C
WT
nog1-R
nog1dis

Fig. S1. Generation of the *nog1dis* mutant. A) Schematic of the construct designed to disrupt the endogenous *NOG1* gene. Primer pairs used to confirm the absence of *NOG1* expression have been indicated with arrows. B) RT-PCR showing the absence of the *NOG1* transcript in the *Ppnog1dis* mutant. C) Representative images of 6-week-old Villersexel wild type, *nog1-R* and *nog1dis* plants showing the presence (wild type) and absence (*nog1-R* and *nog1dis*) of gametophores. Scale bars, 1 cm.

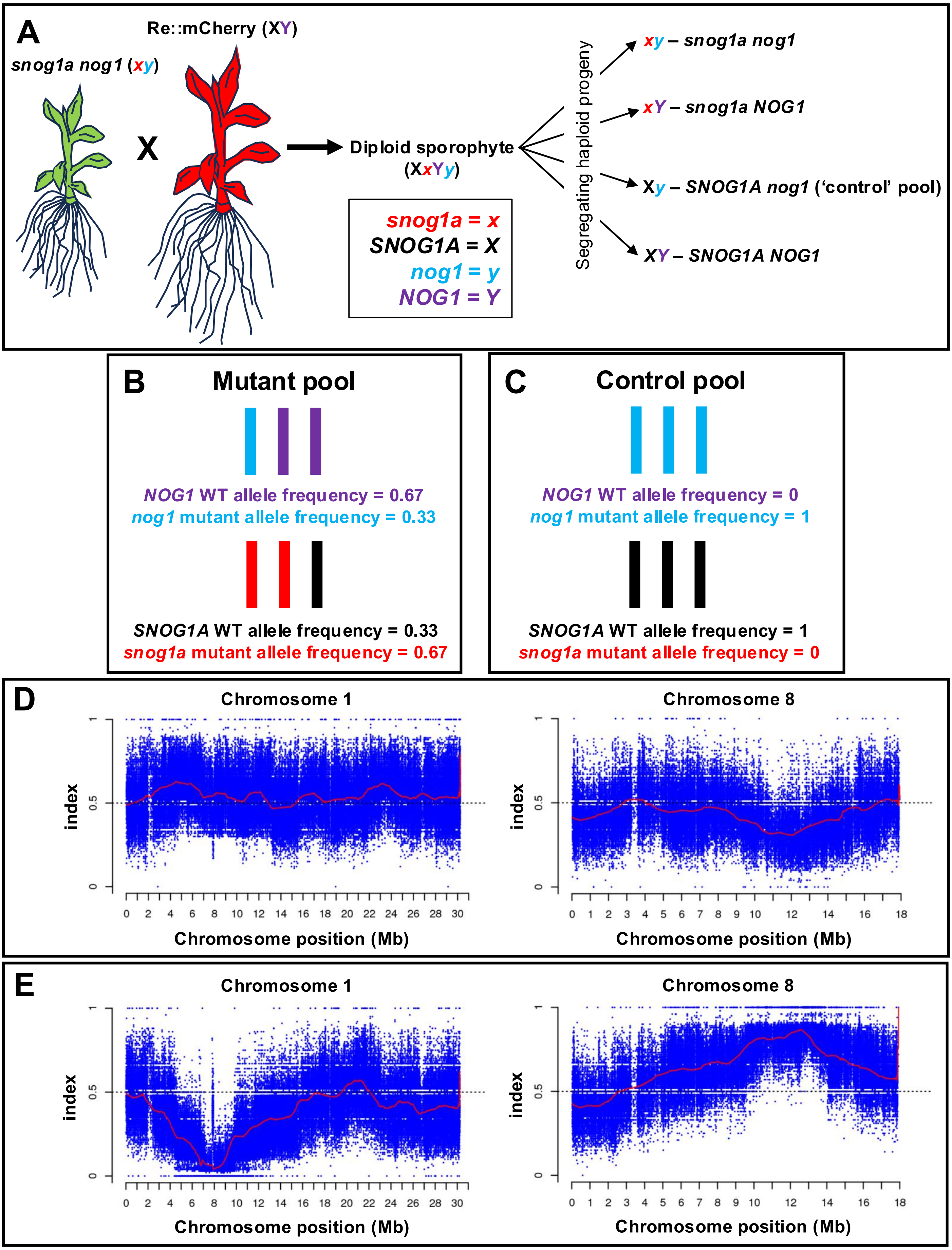
A
Re::mCherry (XY)
snog1a nog1 (xy)
X
Diploid sporophyte (XxYy)
snog1a = x
SNOG1A = X
nog1 = y
NOG1 = Y
Segregating haploid progeny
xy – snog1a nog1
xY – snog1a NOG1
Xy – SNOG1A nog1 (‘control’ pool)
XY – SNOG1A NOG1
B
Mutant pool
NOG1 WT allele frequency = 0.67
nog1 mutant allele frequency = 0.33
SNOG1A WT allele frequency = 0.33
snog1a mutant allele frequency = 0.67
C
Control pool
NOG1 WT allele frequency = 0
nog1 mutant allele frequency = 1
SNOG1A WT allele frequency = 1
snog1a mutant allele frequency = 0
D
Chromosome 1
Chromosome 8
index
Chromosome position (Mb)
E
Chromosome 1
Chromosome 8
index
Chromosome position (Mb)

Fig. S2. Bulk segregant analysis and the identification of the causative mutation in the *snog1a* mutant. A) An outcrossing event between *snog1a* and the Reute::mCherry line yields a diploid sporophyte that undergoes meiosis to produce phenotypically segregating progeny (phenotypic outcomes highlighted). (B,C) Expected *snog1a* mutant, SNOG1A WT, *nog1* mutant and *NOG1* WT allele frequencies in the mutant (B) and control (C) pools respectively. D,E) Plots of the SNP index for the mutant pool (D) and control pool (E) across chromosomes 1 and 8. A SNP index of 0 indicates 100% *nog1dis* parental contribution and a SNP index of 1 indicates 100% Reute::mCherry parental contribution.

Development • Supplementary information

```
Pp3c8_19720_Phytozome    CTCAGCAGCAGATGCAAATGCAAGGCATGCACCCGCCTCCTCAGCATCAACAACTGCAGA 1618
nog1dis                  CTCAGCAGCAGATGCAAATGCAAGGCATGCACCCGCCTCCTCAGCATCAACAACTGCAGA 1618
WT                       CTCAGCAGCAGATGCAAATGCAAGGCATGCACCCGCCTCCTCAGCATCAACAACTGCAGA 1618
snog1a                   CTCAGCAGCAGATGCAAATGCAAGGCATGCACCCGCCTCCTCAGCATCAACAACTGCAGA 1618
                         ************************************************************

Pp3c8_19720_Phytozome    TTCAAGGTACTCCTCAGGCCCCACAGGTGCCGCCGCAAAATGCTCAGTCCCAGCCTCCTC 1678
nog1dis                  TTCAAGGTACTCCTCAGGCCCCACAGGTGCCGCCGCAAAATGCTCAGTCCCAGCCTCCTC 1618
WT                       TTCAAGGTACTCCTCAGGCCCCACAGGTGCCGCCGCAAAATGCTCAGTCCCAGCCTCCTC 1618
snog1a                   TTCAAGGTACTCCTTAGGCCCCACAGGTGCCGCCGCAAAATGCTCAGTCCCAGCCTCCTC 1618
                         ************** *********************************************

Pp3c8_19720_Phytozome    CGCAACCCCATCTTCATCACCAACAATTACAGCTGCAGGCGCCAACTGTAAATCAGCCGG 1738
nog1dis                  CGCAACCCCATCTTCATCACCAACAATTACAGCTGCAGGCGCCAACTGTAAATCAGCCGG 1618
WT                       CGCAACCCCATCTTCATCACCAACAATTACAGCTGCAGGCGCCAACTGTAAATCAGCCGG 1618
snog1a                   CGCAACCCCATCTTCATCACCAACAATTACAGCTGCAGGCGCCAACTGTAAATCAGCCGG 1618
                         ************************************************************

Pp3c8_19720_Phytozome    ATTCTCAGTACCAAACTCAGCAAGCGCCACCGGTTGCTTCTTCACATTCCTCGCAAGTTC 1798
nog1dis                  ATTCTCAGTACCAAACTCAGCAAGCGCCACCGGTTGCTTCTTCACATTCCTCGCAAGTTC 1618
WT                       ATTCTCAGTACCAAACTCAGCAAGCGCCACCGGTTGCTTCTTCACATTCCTCGCAAGTTC 1618
snog1a                   ATTCTCAGTACTAAACTCAGCAAGCGCCACCGGTTGCTTCTTCACATTCCTCGCAAGTTC 1618
                         *********** ************************************************

Pp3c8_19720_Phytozome    CATCTTATTATGCCCAGCAACAGCAACTGCAGCCTGGACAAACAGGCCCAACTCCAGCCA 1858
nog1dis                  CATCTTATTATGCCCAGCAACAGCAACTGCAGCCTGGACAAACAGGCCCAACTCCAGCCA 1618
WT                       CATCTTATTATGCCCAGCAACAGCAACTGCAGCCTGGACAAACAGGCCCAACTCCAGCCA 1618
snog1a                   CATCTTATTATGCCCAGCAACAGCAACTGCAGCCTGGACAAACAGGCCCAACTCCAGCCA 1618
                         ************************************************************
```

Fig. S3. Alignment of *Pp3c8_19720* genomic DNA sequences. An alignment was performed with Pp3c8_19720 genomic DNA sequences from Phytozome (theoretical), and those cloned and sequenced from *nog1dis*, *snog1a* and Reute::mCherry (WT). The SNPs identified in *snog1a* are indicated in red bold type and highlighted in yellow.

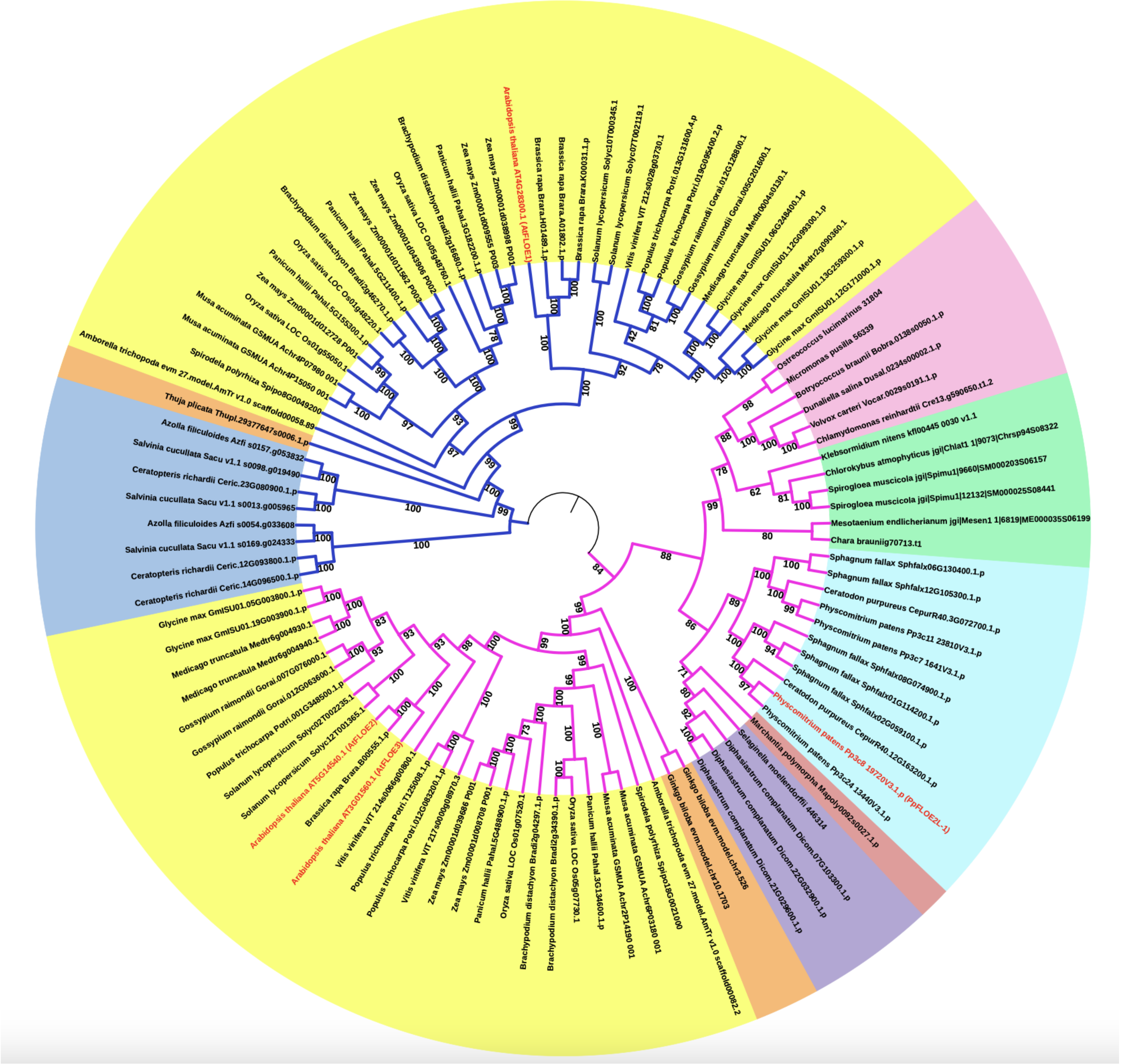

Development • Supplementary information

Fig. S4. Phylogenetic analysis of FLOE-related homologues in the green lineage. Bootstrap values have been indicated on each branch. Both FLOE1L and FLOE2L clades have also been indicated. The highlighted regions denote different groups as follows: chlorophyte algae (pink), charophyte algae (green), mosses (blue), liverworts (red), lycophytes (purple), gymnosperms (orange) and angiosperms (yellow). FLOE2L-1 has been indicated in red text, as well as Arabidopsis FLOE1, FLOE2 and FLOE3.

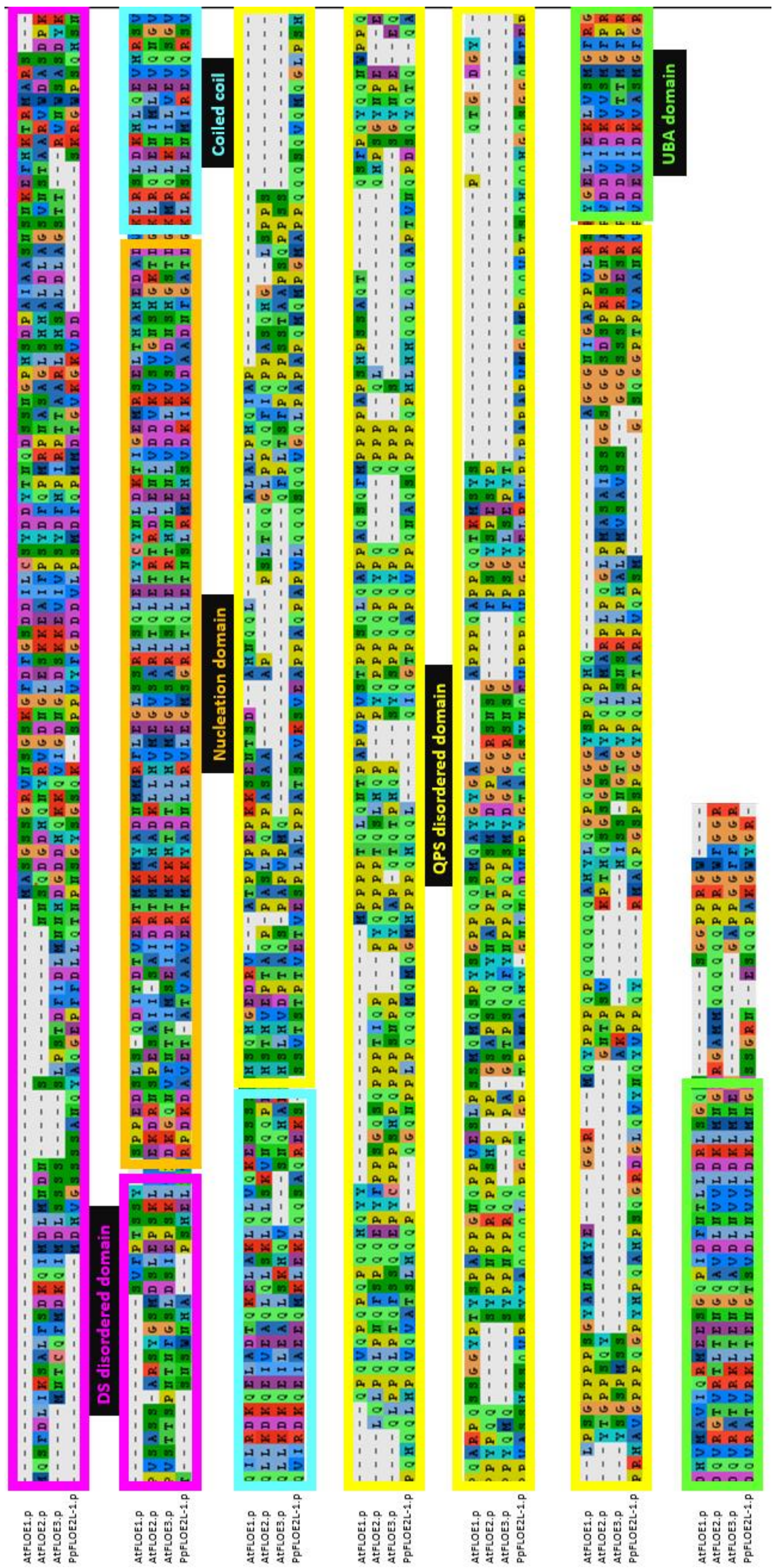

Fig. S5. Alignment of FLOE1, FLOE2, FLOE3 and PpFLOE2L-1. Conserved domains have been highlighted as indicated.

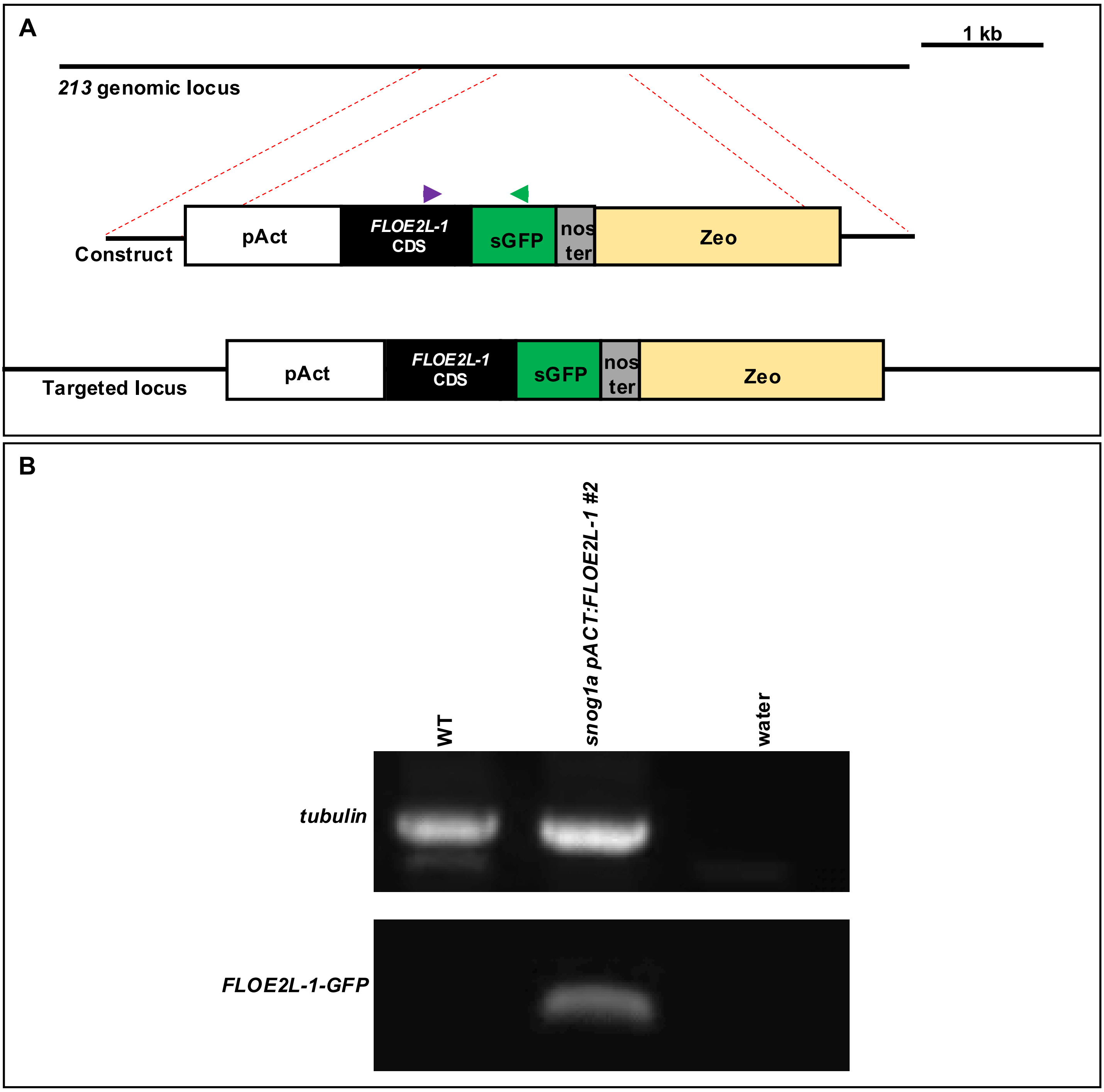

Fig S6. Generation of the *snog1a* complementation line. A) Schematic of the construct used to complement the *snog1a* mutant phenotype, and the resulting targeted locus. B) Genotyping of the complementation line using SNOG1AGFP_F3 and SNOG1AGFP_R3 primers denoted by purple and green arrows in (A) respectively. The construct is only detected in the complemented line and not in wild type (tubulin – control).

Development • Supplementary information

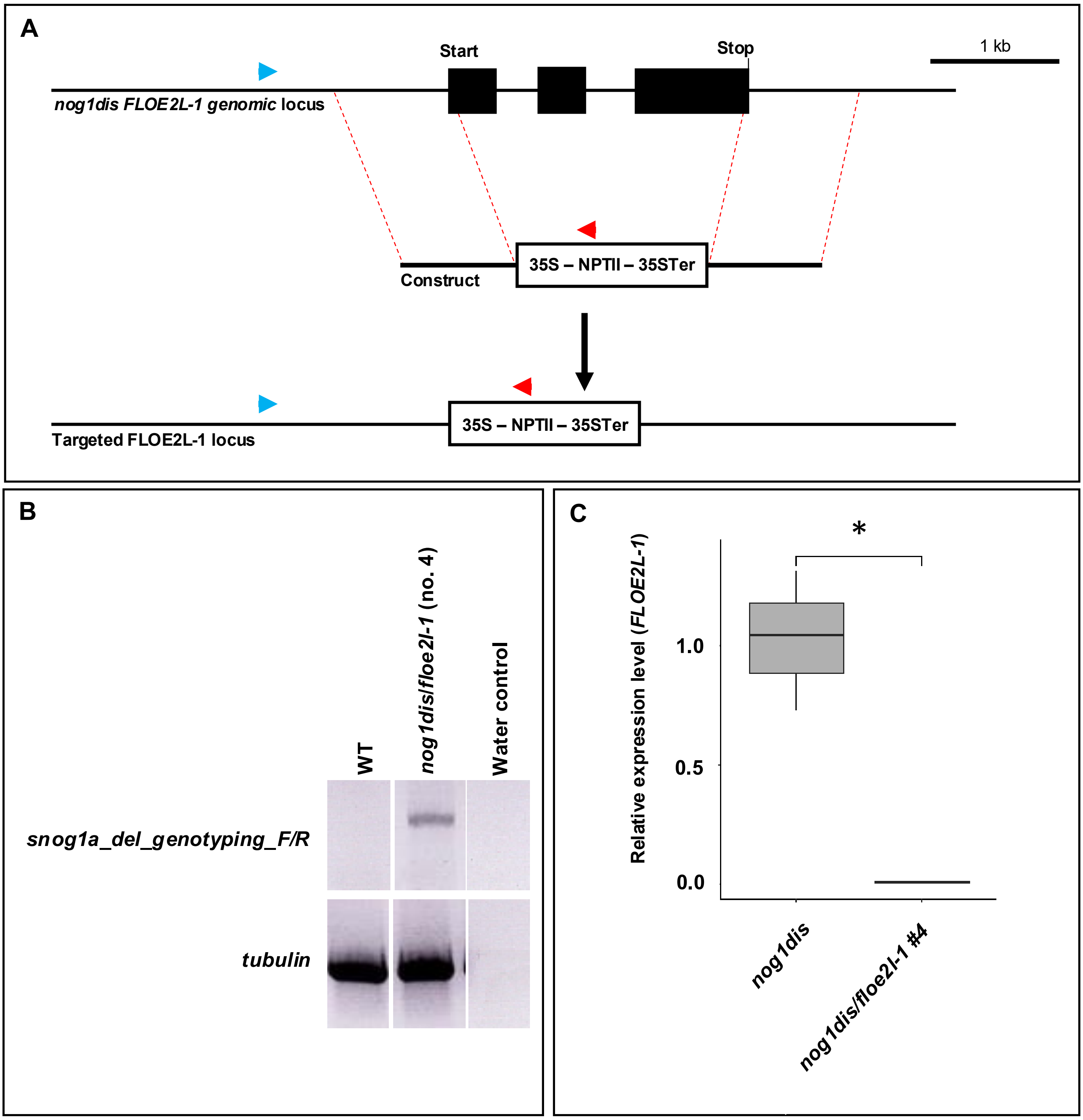

Fig. S7. Generation of the *nog1/floe2l-1* double disruptant line. (A) Schematic of the construct used to disrupt the *FLOE2L-1* locus in the *nog1dis* mutant, and the resulting targeted locus. B) Genotyping of the complementation line using snog1a_del_genotyping_F and snog1a_del_genotyping_R primers denoted by blue and red arrows in (A) respectively (tubulin – control). C) Relative transcript levels of *FLOE2L-1* in *nog1dis* and the *nog1dis/floe2l-1* double disruptant mutant (t test *$p<0.05$).

Development • Supplementary information

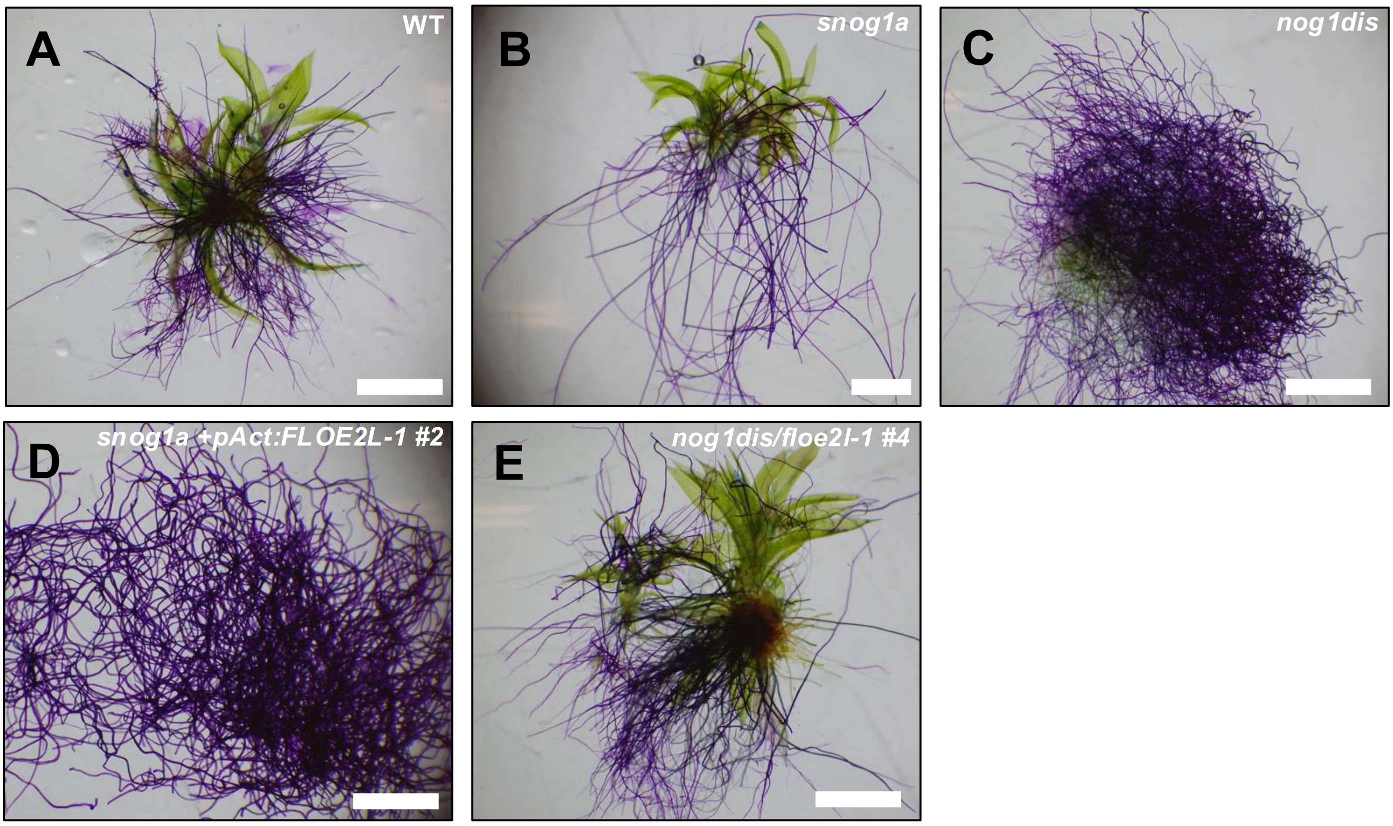

Fig. S8. The *snog1a* mutant can form a fully functional cuticle. (A-E) Toluidine blue staining of 2-month-old wild type (A), *snog1a* (B), *nog1dis* (C), *snog1a* complemented with wild-type *FLOE2L-1* (D) and the *nog1dis/floe2l-1_4* double disruptant (E). Scale bars, 1 mm.

Development • Supplementary information

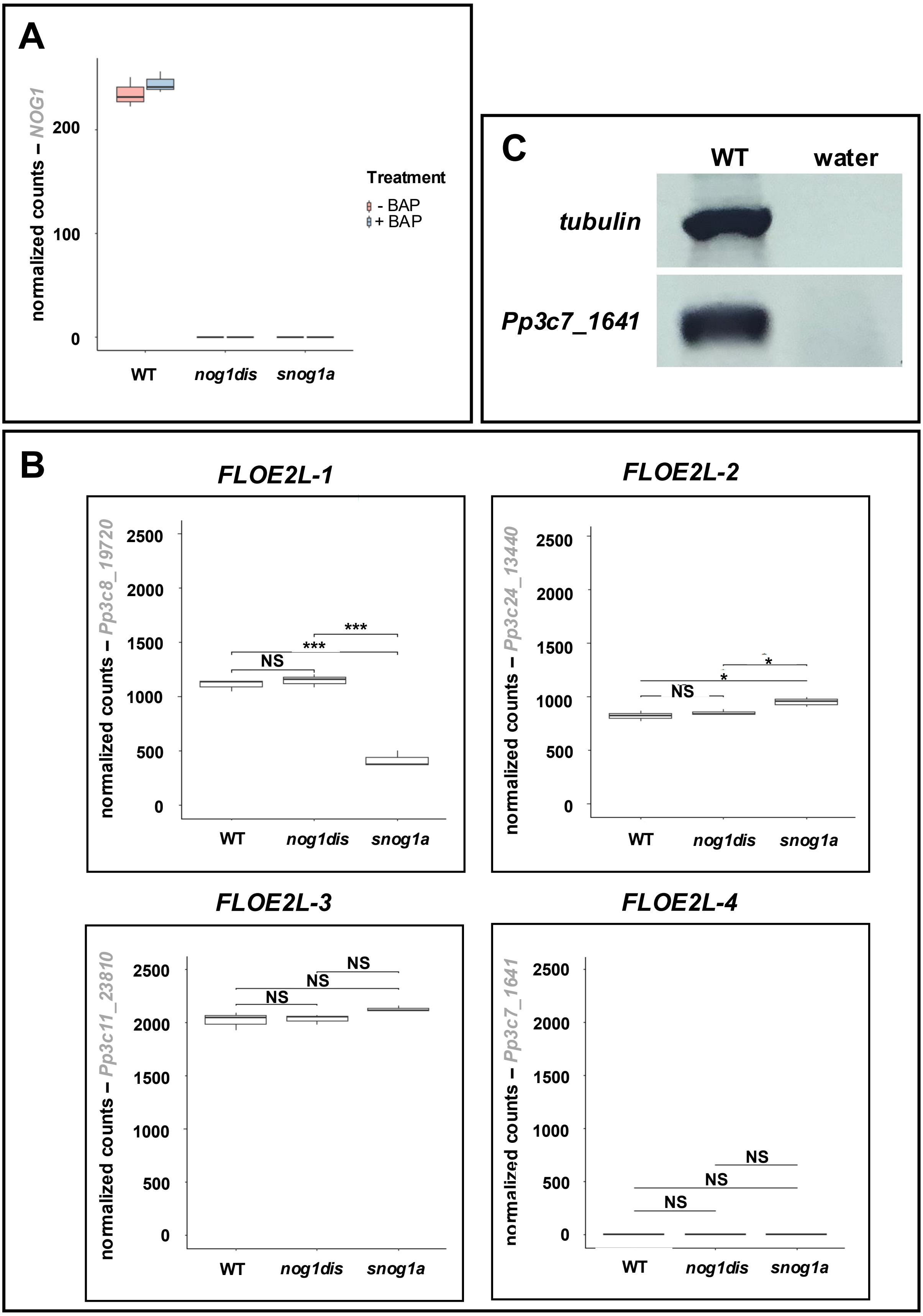
A
normalized counts – NOG1
0
100
200
Treatment
- BAP
+ BAP
WT
nog1dis
snog1a
C
WT
water
tubulin
Pp3c7_1641
B
FLOE2L-1
normalized counts – Pp3c8_19720
2500
2000
1500
1000
500
0
NS
***
***
WT
nog1dis
snog1a
FLOE2L-2
normalized counts – Pp3c24_13440
2500
2000
1500
1000
500
0
NS
*
*
WT
nog1dis
snog1a
FLOE2L-3
normalized counts – Pp3c11_23810
2500
2000
1500
1000
500
0
NS
NS
NS
WT
nog1dis
snog1a
FLOE2L-4
normalized counts – Pp3c7_1641
2500
2000
1500
1000
500
0
NS
NS
NS
WT
nog1dis
snog1a

Fig. S9. Transcriptome comparisons of wild type, *nog1dis* and the *snog1a* mutant. A) Normalized read counts aligned to the *NOG1* gene from the RNA-seq experiment. No reads aligned to *NOG1* in *nog1dis* or the *snog1a* mutant. B) Normalized read counts for FLOE2L genes from the RNA-seq experiment – *Pp3c8_19720* (*FLOE2L-1*), *Pp3c24_13440* (*FLOE2L-2*), *Pp3c11_23810* (*FLOE2L-3*) and *Pp3c7_1641* (*FLOE2L-4*) (t test *p<0.05, **p<0.01, ***p<0.001). C) RT-PCR showing presence of the *FLOE2L-4* transcript in wild type.

Development • Supplementary information

A

Comparison	upregulated	downregulated	total no. DEGs	% total genes
nog1dis **vs** wild type	752	1110	1862	5.80
snog1a **vs** wild type	1350	1365	2715	8.46
snog1a **vs** *nog1dis*	704	437	1141	3.56

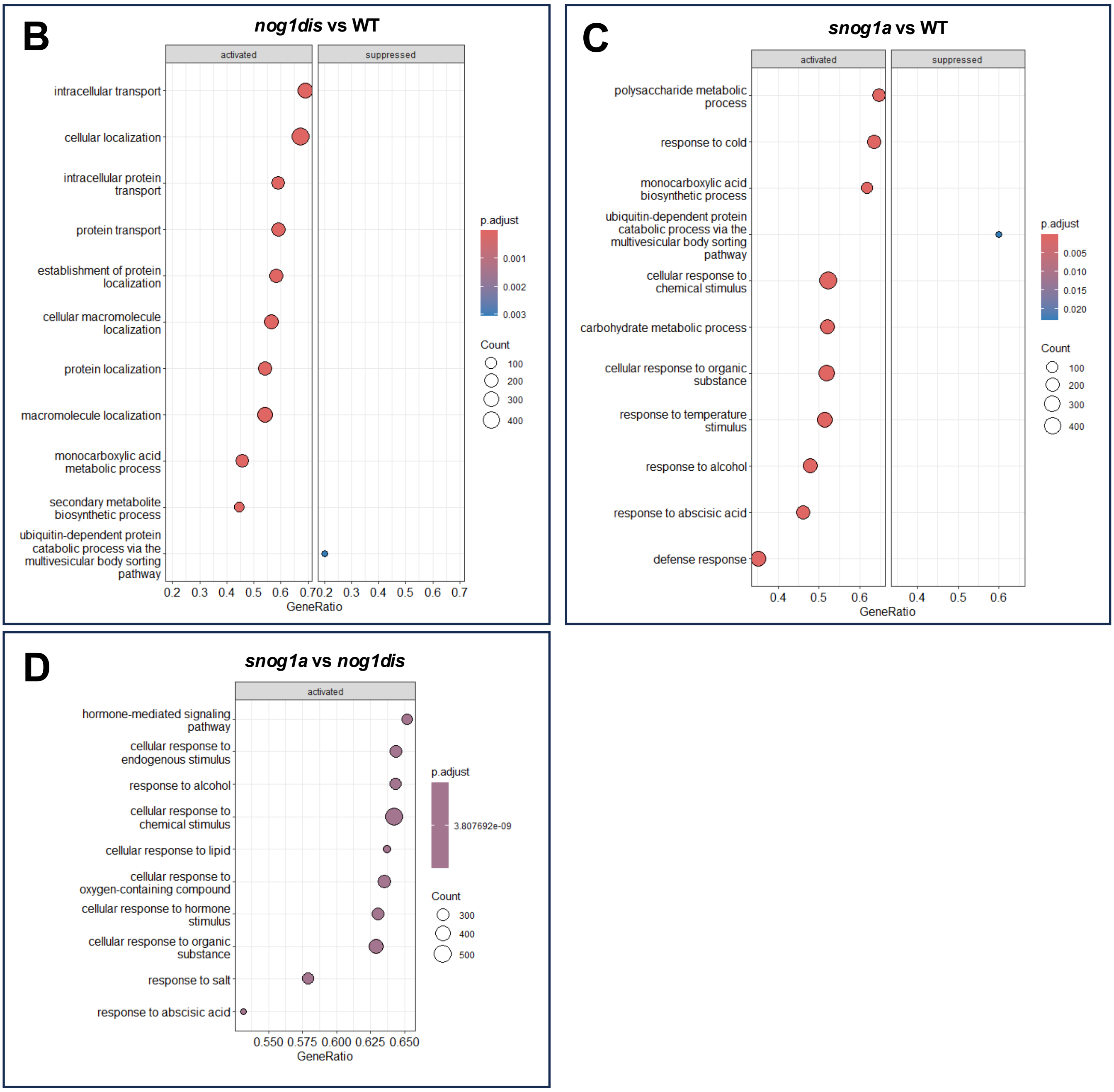

Fig. S10. Differential gene expression analysis for RNA-seq data. A) Table showing results of differential gene expression analysis for RNA-seq data. The number of genes that were significantly differentially expressed (adjusted p value <0.05) in each comparison. B-D) Gene Set Enrichment Analysis (GSEA) with Gene Ontology (GO) biological process terms for *nog1dis* vs wild type (B), *snog1a* vs wild type (C) and *snog1a* vs the *nog1dis* mutant (D). For each comparison, there are up to ten of the most significant activated and suppressed biological processes. The colour indicates the adjusted p-value for the test for enrichment. Count indicated the number of input genes. Gene ratio is the ratio of input genes to the total number of genes in the gene set. Note that because *P. patens* genes were BLASTed again Arabidopsis for this analysis, there were cases where *P. patens* had multiple genes mapping to the same Arabidopsis gene, resulting in some gene ratios exceeding 1.

A

Comparison	upregulated	downregulated	total no. DEGs	% total genes
wild type + BAP **vs** wild type	3417	2723	6140	19.14
nog1dis + BAP **vs** *nog1dis*	274	83	357	1.11
snog1a + BAP **vs** *snog1a*	435	51	486	1.52

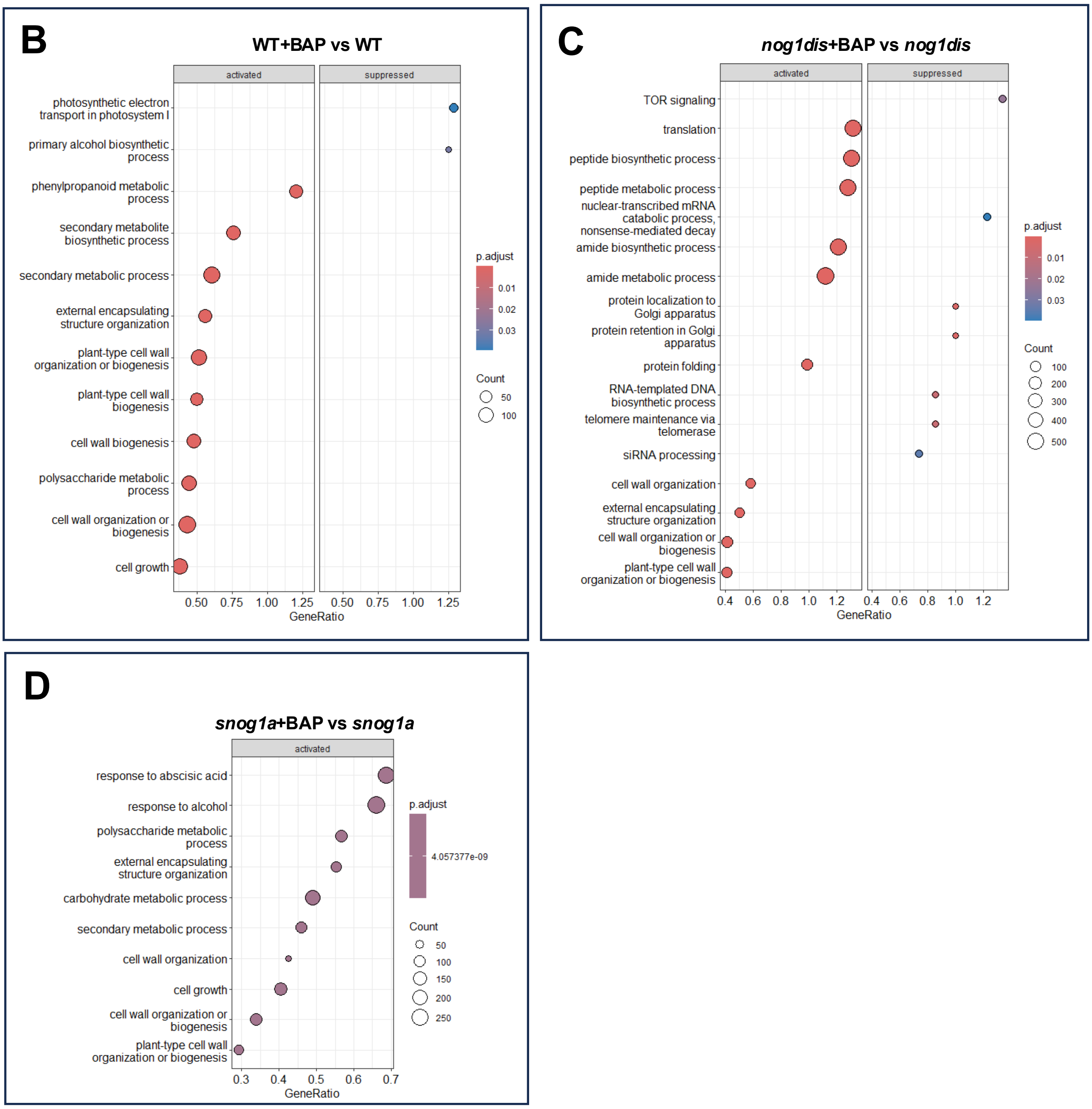

Development • Supplementary information

Fig. S11. Differential gene expression analysis (cytokinin response). A) Table showing results of differential gene expression analysis for RNA-seq data – cytokinin treated compared to control samples. B-D) Gene Set Enrichment Analysis (GSEA) with Gene Ontology (GO) biological process terms for wild type (B), *nog1dis* (C) and the *snog1a* mutant (D) treated in the presence or absence of cytokinin (BAP). For each comparison, there are up to ten of the most significant activated and suppressed biological processes. The colour indicates the adjusted p-value for the test for enrichment. Count indicated the number of input genes. Gene ratio is the ratio of input genes to the total number of genes in the gene set. Note that because *P. patens* genes were BLASTed again Arabidopsis for this analysis, there were cases where *P. patens* had multiple genes mapping to the same Arabidopsis gene, resulting in some gene ratios exceeding 1.

Development • Supplementary information

Table S1. List of primers used in this study

**Generation of the Pp*nog1dis mutant***		
NOG1.5FKpnI	AAAGGTACCCCATCCATGCACACAACCAA	Amplification of 5' region upstream of the *PpNOG1* sequence with KpnI restriction site at the 5' end - forward primer
NOG1.5RXhoI	AAACTCGAGCCTCCGCTCCAAACTCCCAC	Amplification of 5' region upstream of the *PpNOG1* sequence with XhoI restriction site at the 5' end - reverse primer
NOG1.3FNotI	AAGCGGCCGCTAATCTGTGTATGAGTTCAG	Amplification of 3' region downstream of the *PpNOG1* sequence with NotI restriction site at the 5' end - forward primer
NOG1.3RnotI	AAGCGGCCGCGAGTTATCTAGTTTTGTGGA	Amplification of 3' region downstream of the *PpNOG1* sequence with NotI restriction site at the 5' end - reverse primer
**Primers for RT-PCR**		
tubF	TGTGCTGTTGGACAATGAG	Amplification of a *tubulin* transcript - forward primer
tubR	ACATCAGATCGAACTTGTG	Amplification of a *tubulin* transcript - reverse primer
NOG1_GSP.F	GTTGTAGGTTTGGAGTGGCG	Amplification of the *PpNOG1* transcript - forward primer
NOG1_GSP.R	GCAAGTTGAAAAGCCCACCT	Amplification of the *PpNOG1* transcript - reverse primer
NOG1_exon3F	CCCGGAGCTTATTTCAGTTCG	Amplification of partial *PpNOG1* transcript - forward primer
NOG1_exon5R	TGATATTGCTTGCTCCTCATCA	Amplification of partial *PpNOG1* transcript - reverse primer
Pp3c7_1641_qPCR_F	TGCAACGATACCAGGTCCAT	Amplification of *Pp3c7_1641* (*PpFLOE2L-4*) gene for RT-PCR - forward primer
Pp3c7_1641_qPCR_R	ATAAGCGAGAACTCCAGGGC	Amplification of *Pp3c7_1641* (*PpFLOE2L-4*) gene for RT-PCR - reverse primer
***snog1a* mutation verification**		
Pp3c8_19720_int_F	CAAGGCCTACCGTCTCATCC	Amplification of a region within the *Pp3c8_19720* genomic sequence containing the UV-induced mutations - forward primer
Pp3c8_19720_int_R	GTGGAGGAGGGACCTCTTGA	Amplification of a region within the *Pp3c8_19720* genomic sequence containing the UV-induced mutations - reverse primer
**Generation of *snog1a* complementation lines**		
Pp3c8_19720.FSalI	aaagtcgacATGGATCATGTGGGATCC	Amplification of full-length cDNA transcript (no stop codon) with SalI restriction site at 5' end - forward primer
Pp3c8_19720.R_NOSTOP_HindIII	aaaaagcttCCGGCCATACCAGC	Amplification of full-length cDNA transcript (no stop codon) with HindIII restriction site at 5' end - reverse primer
**Verification of *snog1a* complementation lines**		
SNOG1AGFP_F3	GCAAGCATGGGGTTTGGAAG	Amplification of portion of the *Pp3c8_19720-GFP* sequence from genomic DNA - forward primer (in *Pp3c8_19720* CDS sequence)
SNOG1AGFP_R3	GCTGAACTTGTGGCCGTTTA	Amplification of portion of the *Pp3c8_19720-GFP* sequence from genomic DNA - reverse primer (in GFP CDS)
**Verification of *PpFLOE2L-1* disruption lines**		
snog1a_del_genotyping_F	GTCCACCAAGACCACGAAAC	Confirmation of 5' integration at the PpFLOE2L-1 locus - forward primer
snog1a_del_genotyping_R	CATCAGAGCAGCCGATTGTC	Confirmation of 5' integration at the PpFLOE2L-1 locus - reverse primer
**Primers for qPCR**		
Pp3c8_19720_qPCR_F2	CAGCAGCAATCACAGGTTCA	Amplification of *Pp3c8_19720* transcript for qPCR - forward primer
Pp3c8_19720_qPCR_R2	TTCTGTCCTTGCGGTTGTTG	Amplification of *Pp3c8_19720* transcript for qPCR - reverse primer
E2_qPCR_F4	TACGGACCCTAATCCAGATGAC	Amplification of a E2 transcript for qPCR - forward primer
E2_qPCR_R4	CAACCCATTGCATACTTCTGAG	Amplification of a E2 transcript for qPCR - reverse primer

Development • Supplementary information
